# Recombination-Induced Tag Exchange (RITE) Cassette Series to Monitor Protein Dynamics in *Saccharomyces cerevisiae*

**DOI:** 10.1534/g3.113.006213

**Published:** 2013-08-01

**Authors:** Marit Terweij, Tibor van Welsem, Sjoerd van Deventer, Kitty F. Verzijlbergen, Victoria Menendez-Benito, David Ontoso, Pedro San-Segundo, Jacques Neefjes, Fred van Leeuwen

**Affiliations:** * Division of Gene Regulation, Netherlands Cancer Institute, Netherlands Proteomics Centre, 1066CX Amsterdam, The Netherlands; † Division of Cell Biology, Netherlands Cancer Institute, Netherlands Proteomics Centre, 1066CX Amsterdam, The Netherlands; ‡ Instituto de Biología Funcional y Genómica, Consejo Superior de Investigaciones Científicas and University of Salamanca, 37007 Salamanca, Spain

**Keywords:** pulse-chase, epitope tag, protein turnover, protein inheritance

## Abstract

Proteins are not static entities. They are highly mobile, and their steady-state levels are achieved by a balance between ongoing synthesis and degradation. The dynamic properties of a protein can have important consequences for its function. For example, when a protein is degraded and replaced by a newly synthesized one, posttranslational modifications are lost and need to be reincorporated in the new molecules. Protein stability and mobility are also relevant for the duplication of macromolecular structures or organelles, which involves coordination of protein inheritance with the synthesis and assembly of newly synthesized proteins. To measure protein dynamics, we recently developed a genetic pulse-chase assay called recombination-induced tag exchange (RITE). RITE has been successfully used in *Saccharomyces cerevisiae* to measure turnover and inheritance of histone proteins, to study changes in posttranslational modifications on aging proteins, and to visualize the spatiotemporal inheritance of protein complexes and organelles in dividing cells. Here we describe a series of successful RITE cassettes that are designed for biochemical analyses, genomics studies, as well as single cell fluorescence applications. Importantly, the genetic nature and the stability of the tag switch offer the unique possibility to combine RITE with high-throughput screening for protein dynamics mutants and mechanisms. The RITE cassettes are widely applicable, modular by design, and can therefore be easily adapted for use in other cell types or organisms.

Epitope tags provide powerful tools to study protein properties. In general, epitope tags provide a static snapshot of proteins in the cell. However, most proteins are dynamic, and this is often an important aspect of their function (Russel *et al.* 2009; Hinkson and Elias 2011). For example, protein dynamics can influence the mobility and inheritance of proteins, the exchange of subunits of macromolecular complexes, access to otherwise-occupied interaction sites of proteins or, when proteins are degraded and replaced by new ones, in resetting posttranslational modifications (Hager *et al.* 2009; Radman-Livaja *et al.* 2011; Hotz *et al.* 2012; Menendez-Benito *et al.* 2013). During the past few years, several techniques have been developed to measure or visualize protein dynamics. Some of these techniques, such as FRAP (fluorescence recovery after photobleaching), TimeStamp, or derivatives thereof, make use of fluorescent fusion proteins to follow the movement or stability and synthesis of proteins in single cells (Lin and Tsien 2010; Butko *et al.* 2012). Other methods involve differential labeling of old and new proteins by using SILAC (Stable Isotope Labeling with Amino Acids in Cell Culture), radioactive labels, or labeling of specific proteins by using SNAP tags or FlAsH-ReAsH technology (Jansen *et al.* 2007; Adams and Tsien 2008; Sweet *et al.* 2010; Zee *et al.* 2010; Ray-Gallet *et al.* 2011). Most of these methods allow detection of old and new proteins, but only few methods provide the opportunity to specifically purify old and/or newly synthesized proteins by biochemical methods. This aspect is particularly relevant for the study of chromatin protein dynamics, where affinity purification allows mapping of protein occupancy and dynamics on specific regions of the genome.

Several methods recently have been developed to measure chromatin protein dynamics (recently reviewed in Deal and Henikoff 2010a). One is the use of inducible overexpression of a tagged version of the protein of interest in the presence of an endogenously expressed untagged (or differentially tagged) copy (Korber *et al.* 2004; Dion *et al.* 2007; Jamai *et al.* 2007; Kim *et al.* 2007; Rufiange *et al.* 2007). Another method (CATCH-IT; covalent attachment of tags to capture histones and identify turnover) involves the labeling of newly synthesized proteins by amino acid analogs that can be coupled to biotin and thereby used for selective purification (Deal and Henikoff 2010a,b). In *Physarum*, the dynamics of histone proteins can be monitored at the single-cell level by microinjection of small amounts of labeled histone proteins (Thiriet and Hayes 2005; Ejlassi-Lassallette *et al.* 2011). We recently developed a versatile and flexible method called recombination-induced tag exchange (RITE), in which epitope tags on an endogenous protein of interest can be swapped in a conditional manner by an inducible Cre recombinase (Verzijlbergen *et al.* 2010; De Vos *et al.* 2011; Radman-Livaja *et al.* 2011; Hotz *et al.* 2012).

The RITE system has been developed in budding yeast and is composed of two parts; a tandem-tag cassette that can be integrated behind the gene of interest for conditional C-terminal tagging, and a stably integrated and constitutively expressed hormone-dependent Cre recombinase that allows control of epitope switching. RITE cassettes encode for one epitope tag (Tag 1 or old tag) between two LoxP recombination sites and a second, orphan, epitope tag (Tag 2 or new tag) downstream of the second LoxP site (Figure 1A). Upon activation of Cre recombinase activity by the simple addition of the hormone estradiol, a tag switch occurs: the first tag is removed from the genome by recombination between the two LoxP sites and replaced by the second tag. To prevent background recombination, the RITE cassettes contain a selectable marker between the LoxP sites. Note that the LoxP recombination sequence is part of the protein coding sequence in the RITE cassettes, resulting in an in-frame tag following the LoxP sites and allowing for switching by recombination.

**Figure 1:**
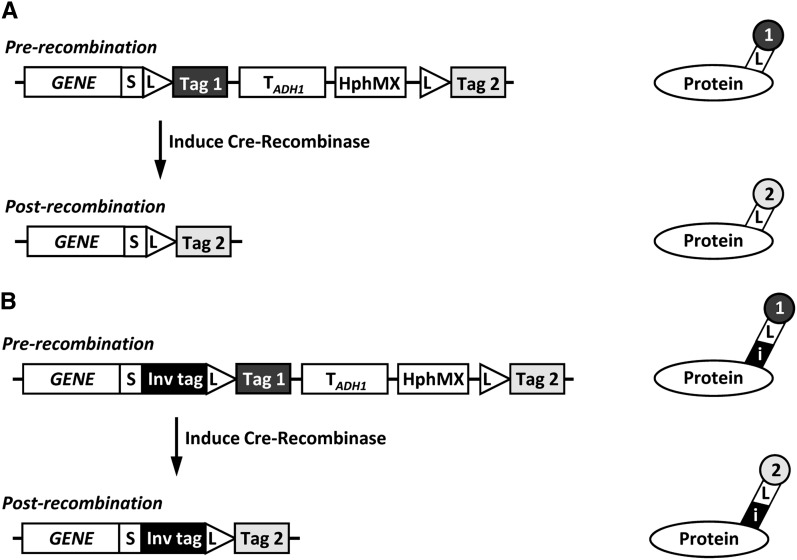
Outline of RITE. (A) After integration of a RITE cassette behind the gene of interest (*GENE*), recombination between LoxP sites is induced by Cre-Recombinase, causing a permanent switch from old Tag 1 to new Tag 2 on the protein of interest. S, spacer; L, LoxP recombination sites; T*_ADH1_*, *ADH1* terminator; HphMX, Hygromycin resistance cassette. (B) RITE cassette including an invariant tag (i) upstream of the first LoxP site. The invariant tag is present pre- and postrecombination and can be used for simultaneous detection of the old and new protein of interest.

RITE has several advantages over some of the other methods to measure protein dynamics. The proteins of interest are expressed form their endogenous promoter, avoiding potential problems with overexpression. In addition, a RITE switch does not require specific media changes and is permanent, which allows monitoring of protein dynamics under many different physiological conditions (De Vos *et al.* 2011; Radman-Livaja *et al.* 2011; Hotz *et al.* 2012; Ouellet and Barral 2012; Menendez-Benito *et al.* 2013). Importantly, old and newly synthesized proteins can be monitored simultaneously. The RITE system is flexible, widely applicable, and compatible with switching between different short epitope tags as well as fluorescent tags. Furthermore, RITE allows selective tagging and following one protein of interest in the context of all other unlabeled protein. Finally, RITE allows dynamics measurements in the context of a genetic screen, allowing identification of proteins controlling protein turnover (Verzijlbergen *et al.* 2011; Hotz *et al.* 2012). Of note, proteins that are subject to very high turnover may not be suitable for analysis by RITE because Cre-induced recombination of the LoxP sites takes several hours to complete (see *Results and Discussion* and Verzijlbergen *et al.* 2010).

Here we present a comprehensive toolbox for the RITE assay. The availability of a RITE cassette series containing diverse biochemical and fluorescent tags allows for selection of tag pairs that are optimal for the protein of interest or for the experimental setup. Furthermore, new RITE cassettes with additional invariant tags are presented that bypass the need for protein-specific antibodies and allow for simultaneous detection of old and new protein (Figure 1B). The RITE cassettes are modular by design. Therefore, they can be easily adapted to modify tags or to adjust cassettes for use in other cell types or organisms for which inducible Cre recombinases are available.

## Materials and Methods

### Strains and growth conditions

Yeast and bacteria were cultured under standard conditions (van Leeuwen and Gottschling 2002). *Escherichia coli* strain DH5α was used for plasmid preparations. All yeast strains constructed and used in this study are detailed in Table 1 and were derived from previously published strains (Brachmann *et al.* 1998; van Leeuwen *et al.* 2002; Tong and Boone 2006; Verzijlbergen *et al.* 2010; De Vos *et al.* 2011; Verzijlbergen *et al.* 2011). Strains were grown in YEPD (1% yeast extract, 2% bacto peptone, 2% glucose) in shaking flasks at 30°. To select for Hygromycin resistance, cells were grown in YEPD containing 200 µg/mL Hygromycin B (Invitrogen).

**Table 1:** Yeast strains

Name	Relevant Genotype	Switch Parent	Source*^a^*
NKI2036*^b^*	MAT**a** *his3Δ1 leu2Δ0 lys2Δ0 met15Δ0 ura3Δ0 hhf1-hht1Δ*::*LEU2*		KV
NKI2148	NKI2036; *bar1Δ*::*HisG his3Δ1*::*HIS3*-P*_TDH3_-CRE-EBD78 hht2*::*HHT2-LoxP-HA*-T*_ADH1_-HphMX-LoxP-T7*		KV
NKI8085	NKI2036; *hht2*::*HHT2-LoxP-T7*	NKI2148	This study
NKI2158	NKI2036; T*_CYC1_*::P*_TDH3_-CRE-EBD78-HIS3 hht2*::*HHT2-LoxP-T7*-T*_ADH1_-HphMX-LoxP-HA*		KV
NKI4138	NKI2036; *hht2*::*HHT2-LoxP-HA*	NKI2158	This study
BY4733	MAT**a** *his3Δ200 leu2Δ0 met15Δ0 trp1Δ63 ura3Δ0*		CB
NKI8001	BY4733; *bar1Δ*::*HISG his3*::*HIS3*-P*_TDH3_-CRE-EBD78*		This study
NKI2176	BY4733; *hht1-hhf1*::*MET15 bar1Δ*::*HisG his3Δ200*::*HIS3*-P*_TDH3_-CRE-EBD78*		This study
NKI2178	NKI2176; *hht2*::*HHT2-LoxP-HA-6xHis*-T*_ADH1_-HphMX-LoxP-T7*		This study
NKI8086	NKI2176; *hht2*::*HHT2-LoxP-T7*	NKI2178	This study
NKI2220	NKI2176; *hht2*::*HHT2-LoxP-T7*- T*_ADH1_-HphMX-LoxP-HA-6xHis*		This study
NKI8037	NKI2220; *hht2*::*HHT2-LoxP-HA-6xHis*	NKI2220	This study
NKI8051	NKI2176; *hht2*::*HHT2-LoxP-2xT7*- T*_ADH1_-HphMX-LoxP-HA-6xHis*		This study
NKI8088	NKI8051; *hht2*::*HHT2-LoxP-HA-6xHis*	NKI8051	This study
NKI8056	NKI2176; *hht2*::*HHT2-LoxP-HA-6xHIS*- T*_ADH1_-HphMX-LoxP-V5*		This study
NKI8058	NKI8056; *hht2*::*HHT2-LoxP-V5*	NKI8056	This study
NKI8050	NKI2176; *hht2*::*HHT2-LoxP-V5*- T*_ADH1_-HphMX-LoxP-HA-6xHis*		This study
NKI8087	NKI8050; *hht2*::*HHT2-LoxP-HA-6xHis*	NKI8050	This study
NKI8052	NKI2176; *hht2*::*HHT2-LoxP-2xFLAG*- T*_ADH1_-HphMX-LoxP-HA-6xHis*		This study
NKI8089	NKI8052; *hht2*::*HHT2-LoxP-HA-6xHis*	NKI8052	This study
NKI8030	NKI8001; *htz1*::*HTZ1-LoxP-HA-6xHIS*-T*_ADH1_-HphMX-LoxP-T7*		
NKI8053	NKI8001; *htz1*::*HTZ1-LoxP-V5*-T*_ADH1_-HphMX-LoxP-HA-6xHIS*		
NKI8054	NKI8001; *htz1*::*HTZ1-LoxP-2xT7*-T*_ADH1_-HphMX-LoxP-HA-6xHIS*		
NKI8055	NKI8001; *htz1*::*HTZ1-LoxP-2xFLAG*-T*_ADH1_-HphMX-LoxP-HA-6xHIS*		
NKI4044	BY4733; *pgk1*::*PGK1-V5-LoxP-HA-yEGFP*- T*_ADH1_-HphMX-LoxP-T7-mRFP*		This study
NKI4044post	NKI4044; *pgk1*::*PGK1-LoxP-T7-mRFP*	NKI4044	This study
Y7092	MAT**α** *can1Δ*::P*_STE2_-Sp-his5 lyp1Δ his3Δ1 leu2Δ0 ura3Δ0 met15Δ0*		AT
SPC42G	Y7092; *lyp1Δ*::*NATMX*-P*_TDH3_-CRE-EBD78 spc42*::*SPC42-loxP-T7*- T*_ADH1_-HphMX-loxP-HA-GFP*		This study
SPC42Gpost	SPC42G; *spc42*::*SPC42-loxP-HA-GFP*	SPC42G	This study
SPC42R	Y7092; *lyp1Δ*::*NATMX*-P*_TDH3_-CRE-EBD78 spc42*::*SPC42-LoxP-3xHA*- T*_ADH1_-HphMX-LoxP-3xT7-mRFP*		This study
SPC42Rpost	SPC42R; *spc42*::*SPC42-LoxP-3xT7-mRFP*	SPC42R	This study

a Sources: Verzijlbergen *et al.* 2010; Brachmann *et al.* 1998; Tong *et al.* 2006; van Leeuwen *et al.* 2002, see *Materials and Methods*.

b NKI2036 was derived from crosses between BY4742, BY4727, and UCC1369 (CB and FvL).

### Construction of RITE cassettes

**Figure 2:**
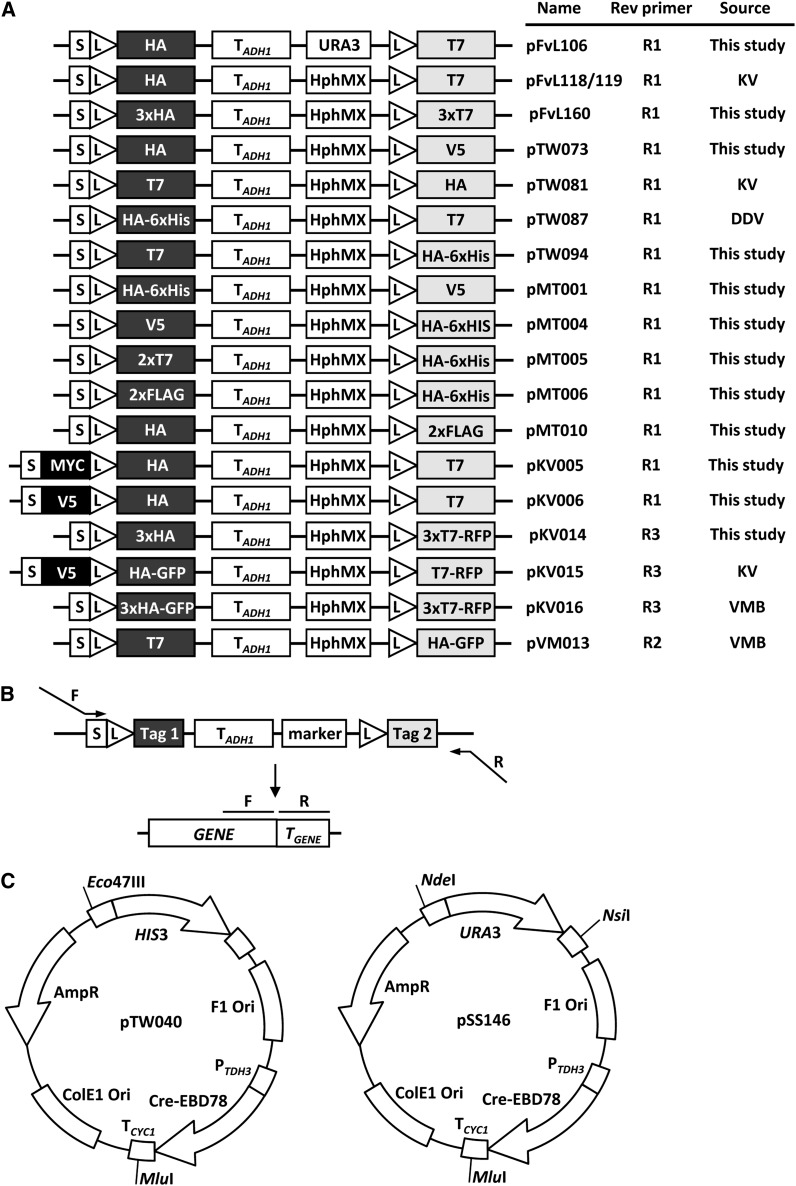
RITE cassettes and Cre recombinase vectors. (A) Available RITE cassettes. The cassettes can be amplified with the primers indicated (see panel B). The sequence of the forward primer F1 is 5′-GGT GGA TCT GGT GGA TCT-3′ (corresponding to the reading frame of the spacer sequence); the sequences of the reverse primers are: R1 5′-AGGGAACAAAAGCTTGCATG-3′; R2 5′-TGATTACGCCAAGCTCG-3′; R3 5′-TCA GGCGCCGGTGGAGTGGCG-3′. Note that primer R3 contains a STOP codon (underlined), because the mRFP sequence in pKV14-16 lacks a stop codon. (B) Gene targeting of RITE cassettes by homologous recombination. The RITE cassettes can be PCR-amplified with a forward and reverse primer that have 20 bp homology with the cassette and a tail of at least 40 bp homology with the 3′ end of the gene of interest and 3′ UTR, respectively. (C) Constructs to integrate the Cre recombinase expression vector in the yeast genome by homologous recombination. Unique restriction sites can be used to digest the plasmid and integrate the linear fragment by single cross-over (see *Materials and Methods*).

All plasmids constructed in this study were derived from the previously described pFvL100 (Verzijlbergen *et al.* 2010) and are listed in Figure 2A and Supporting Information, File S1. pFvL106 contains a cassette switching from an HA (Hemagglutinin) to a T7 epitope tag (HA→T7), but the *HphMX* selection marker (the Hygromycin B phospho transferase gene under control of the Ag*TEF1* promoter and terminator) is replaced by a *URA3* selection marker. pFvL118 and pFvL119 (HA→T7) are nearly identical except for small sequence differences around the *HphMX* gene (Verzijlbergen *et al.* 2010). For pFvL160, oligos were created containing either 2xT7 with *Bsr*GI overhangs or 2xHA with *Spe*I overhangs. Single-stranded oligos (Table 2) of matching tags were incubated in 40 mM Tris, pH 8, and 100 mM NaCl in a thermal cycler set at 94° for 3 min with a gradual cooling down to 15°. The double-stranded fragments were purified over a microspin G-50 column (Amersham Biosciences). Oligos were sequentially cloned into pFvL119 digested with the appropriate restriction enzymes. pTW073 was constructed by amplifying a fragment of pFvL118 with primers containing a V5 tag extension and *Bsr*GI restriction sites. Subsequently, this fragment and pFvL118 were digested with *Bsr*GI and ligated together, creating an HA→V5 cassette.

**Table 2:** Oligos used for plasmid construction

Oligo	Sequence
CLTSB19a	GTACTATGGCTTCTATGACAGGAGGTCAACAGATGGGAGGAATGGCCTCAATGACCGGTGGCCAGCAAATGGGAT
CLTSB19b	GTACATCCCATTTGCTGGCCACCGGTCATTGAGGCCATTCCTCCCATCTGTTGACCTCCTGTCATAGAAGCCATA
CLTSB17a	CTAGCTATCCCTATGACGTCCCGGACTATGCAGGATATCCATATGACGTTCCAGATTACGCTA
CLTSB17b	CTAGTAGCGTAATCTGGAACGTCATATGGATATCCTGCATAGTCCGGGACGTCATAGGGATAG
KpnI-V5-*Spe*I Fwd	CGGTAAGCCTATCCCTAACCCTCTCCTCGGTCTCGATTCTA
KpnI-V5-*Spe*I Rev	CTAGTAGAATCGAGACCGAGGAGAGGGTTAGGGATAGGCTTACCGGTAC
KpnI-2xFLAG-*Spe*I Fwd	CGACTACAAGGACGATGACGATAAAGATTATAAAGATGACGATGACAAGA
KpnI-2xFLAG-*Spe*I Rev	CTAGTCTTGTCATCGTCATCTTTATAATCTTTATCGTCATCGTCCTTGTAGTCGGTAC
KpnI-2xT7-*Spe*I Fwd	CATGGCTTCTATGACAGGAGGTCAACAGATGATGGCAAGCATGACTGGTGGACAGCAAATGA
KpnI-2xT7-*Spe*I Rev	CTAGTCATTTGCTGTCCACCAGTCATGCTTGCCATCATCTGTTGACCTCCTGTCATAGAAGCCATGGTAC

Fwd, forward, Rev, reverse.

A 3-step polymerase chain reaction (PCR) approach was used to construct pTW081 and pTW087. Fragments containing either HA flanked by *Mlu*I and *Sph*I restriction sites, or T7 flanked by *Cla*I and *Nsi*I restriction sites were amplified from pFvL118. These fragments and pFvL118 were digested with the respective restriction enzymes and ligated to construct a cassette with swapped tags (pTW081; T7→HA; Verzijlbergen *et al.* 2010). For pTW087 a fragment containing HA was amplified using primers with a tail of 6xHis, creating an HA-6xHis fragment flanked by *Cla*I and *Nsi*I restriction sites. The fragment and pFvL118 were digested with *Cla*I and *Nsi*I and ligated to construct an HA-6xHis→T7 cassette (pTW087; De Vos *et al.* 2011). An HA-6xHis fragment flanked by *Bgl*II and *Bsr*GI restriction sites was amplified by PCR from pTW087. The fragment and pTW081 were digested with these enzymes and ligated, constructing pTW094 (T7→HA-6xHis). For pMT001 (HA-6xHis→V5), plasmids pTW087 and pTW073 were digested with *Nsi*I and *Mlu*I. The fragment of pTW087 containing spacer-LoxP-HA-6xHis-T*_ADH1_-HphMX* was ligated into the fragment of pTW073 containing LoxP-V5. For pMT004-006 a fragment of double stranded DNA containing V5, 2xT7, or 2xFLAG with *Kpn*I and *Spe*I overhangs were made by oligo annealing (Table 2). Oligos were ligated into pFvL119 digested with *Kpn*I and *Spe*I. The tags of the resulting plasmids were combined with the HA-6xHis tag of pTW094 using *Asc*I and *Sph*I restriction sites to generate pMT004 (V5→HA-6xHIS), pMT005 (2xT7→HA-6xHIS), and pMT006 (2xFLAG→HA-6xHIS).

pKV005 was constructed by a 3-step PCR on pKV001 (a pFvL119 derivative containing additional restriction sites), introducing an invariant MYC tag between the spacer and LoxP site. The resulting cassette is MYCi-HA→T7. For pKV006, a V5-fragment with *Eco*47III restriction sites was amplified by PCR. The fragment and pKV001 were digested by *Eco*47III and ligated, constructing a cassette with an invariant V5 tag between the spacer and LoxP site (V5i-HA→T7). For pKV014, monomeric red fluorescent protein (mRFP) was amplified by PCR with primers containing *Bsr*GI and *Hind*III restriction sites at the ends. This fragment and pFvL160 were digested with *Bsr*GI and *Hin*dIII and ligated, resulting in a cassette with a fluorescent tag post-recombination (3xHA→3xT7-mRFP). For pKV015, yEGFP and mRFP were amplified with primers containing restriction sites for *Spe*I, and for *Bsr*GI and *Hind*III, respectively. These fragments and pKV006 were digested with the appropriate enzymes and ligated, resulting in a cassette that combines epitope tags with fluorescent tags, pKV015 (V5i-HA-yEGFP→T7-mRFP, (Verzijlbergen *et al.* 2010)). For pVM013, pTW081 and pVM012 were digested with *Bsr*GI and *Hind*III. The small fragment of pVM012 containing yEGFP was ligated into the large fragment of pTW081 (T7→HA), resulting in a T7→HA-yEGFP cassette (Menendez-Benito *et al.* 2013).

### PCR-mediated gene tagging

To target RITE cassettes to the gene of interest, the cassette was amplified using integration primers that contain 40 bp of sequence of the gene of interest for homologous recombination (Figure 2B). The forward primer (F1) sequence of the cassette is 5′-GGT GGA TCT GGT GGA TCT-3′. For pFvL106 to pKV006, the reverse primer used to amplify the cassette is 5′-AGGGAACAAAAGCTTGCATG-3′ (R1), which anneals 54 bp downstream of the cassette. For pKV014 to pKV016 the reverse primer sequence of the cassette is 5′-TCAGGCGCCGGTGGAGTGGCG-3′ (R3). This primer is mRFP specific and anneals at the end of mRFP to introduce a stop codon (underlined sequence), which is missing in the mRFP sequence. The reverse primer used for pVM013 is 5′-TGATTACGCCAAGCTCG-3′ (R2), which anneals further downstream than R1 (90 bp downstream of the cassette) and which can be used when the sequence of R1 is absent. For one strain in this study (SPC42G), a reverse primer was used that anneals even further downstream of the cassette. When designing primers to target RITE to the gene of interest, it is important that the reading frame of the ORF and the fused cassette is maintained. The PCR products were transformed into strain NKI2176, NKI2036 or Y7092 using standard transformation protocols (Gietz and Schiestl 2007). After transformation, cells were plated onto YEPD plates and incubated overnight at 30°; the following day plates were replica-plated onto selection plates (YEPD containing 200 µg/mL Hygromycin). Integration of the cassettes was checked by colony PCR.

### Cre recombinase vectors

Two plasmids were constructed to integrate Cre recombinase in the yeast genome. pTW040 was constructed by cloning a P*_TDH3_*-Cre-EBD78 fragment digested with *Pvu*II into pRS303 digested with *Sma*I. For the P*_TDH3_*-Cre-EBD78 fragment, P*_TDH3_* was cloned with *Apa*I and *Bsp*EI upstream of Cre-EBD78, where the P*_GAL_* had been replaced by a multiple cloning site. pSS146 was constructed by cloning the P*_TDH3_*-Cre-EBD78 fragment of pTW040 into pRS306 with *Eco*RI and *Not*I. Both plasmids contain unique restriction sites that can be used to integrate Cre-Recombinase at the *HIS3* locus, the *URA3* locus or the *CYC1* terminator (Figure 2C).

### Detection of recombination by Southern blot

For Southern blotting 5 × 10^8^ cells were spun and frozen at −80°. A histone H3 *(HHT2*)-specific probe was made by PCR amplification using primers HHT2_*Hin*dIII_for (GAATCTTTCTGTGACGCTTGG) and HHT2_*Hin*dIII_rev (GGGGAAGAACAGTTGGAAGG), resulting in a 650-bp amplicon covering the region 576,144–576,794 of chromosome XIV. When used on genomic DNA that was digested with *Hind*III, the three bands recognized are specific for before the switch (3000 bp, pre), after the switch (931 bp, post), or as an internal control (1538 bp, control). Radioactive Southern blotting was performed using 50 μCi of ^32^P-dCTP; incubation occurred overnight at 65°.

### Protein detection by immunoblot and antibodies

For immunoblotting, strains were grown to mid-log phase (OD_660_ 0.6-0.9). Samples of 2 × 10^8^ cells were harvested and washed with TE [10 mM Tris, pH 8; 1 mM ethylenediaminetetraacetic acid (EDTA)] containing 0.2 mM phenylmethylsulfonyl fluoride (PMSF). Cell pellets were stored at −80° until further processing, but at least 30 min. Whole-cell extracts were prepared in SUMEB (1% sodium dodecyl sulfate (SDS); 8 M urea; 10 mM 3-(N-morpholino)propanesulfonic acid (MOPS), pH 6.8; 10 mM EDTA; 0.01% bromophenol blue) containing protease inhibitors (1 mM PMSF, 1 mM dithiothreitol (DTT), 5 mM benzamidine, 1 µg/mL pepstatin, 1 µg/mL leupeptin) by glass bead disruption in a multivortex. The resulting lysate was incubated for 10 min at 65° and subsequently clarified by centrifuging 5 min at 20,817 x g. Before immunoblotting, 4 to 10 µL of lysate was separated on a polyacrylamide gel (16% for histone H3 and H2B, 10% for Pgk1, Spc42, and Sir2). Separated proteins were transferred to a 0.45-µm nitrocellulose membrane for one (H3 and H2B) or two (Pgk1, Spc42, and Sir2) hours at 0.1 A. Membranes were blocked with phosphate-buffered saline (PBS) containing 2% or 5% Nutrilon (Nutricia) for 1 hr, and first antibody incubations were done either for 2 hr at room temperature or overnight at 4° in Tris-buffered saline containing 10% Tween-20 (TBST) with 2% Nutrilon. After washing three times in TBST, secondary antibody incubation was performed in TBST with 2% Nutrilon and LI-COR Odyssey IRDye 800CW antibody at 1:10,000 for 45 min at room temperature in the dark followed by 10-minute washes twice in TBST and once in PBS. Membranes were scanned using a LI-COR Odyssey IR Imager (Biosciences) and analyzed using the Odyssey LI-COR software package version 3.0. Antibodies used in this study are Pgk1 (A-6457, Invitrogen), Sir2 (Sc-6666, Santa Cruz), histone H2B (39238, Active Motif), HA (12CA5), T7 (A190-117A, Bethyl), Flag (M2 F3165, Sigma), V5 (R960-25, Invitrogen), histone H3 and LoxP (Verzijlbergen *et al.* 2010), and green fluorescent protein (GFP) and mRFP (Rocha *et al.* 2009).

### Chromatin immunoprecipitation (ChIP)

For ChIP, cells were grown to mid-log phase in YEPD with 200 µg/mL Hygromycin for preswitch strains or YEPD for postswitch strains. Samples of 1−3 × 10^9^ cells were taken, fixed for 10 min in 1% formaldehyde, and washed with cold TBS. Pellets were stored in 12-mL flat bottom-tubes or 2-mL screw-cap tubes at −80° until further processing. Cells in 12-mL tubes were disrupted in 300 µL of breaking buffer (100 mM Tris, pH 7.9; 20% glycerol; protease inhibitor cocktail EDTA-free; Roche) with 400 µL of glass beads in a multivortex for 20 min at 4°. Cells in screw-cap tubes were disrupted in 200 µL of breaking buffer with 500 µL of glass beads in a bead beater in a 4° cold block for 2 min. Lysis was at least 70%, as determined by microscopy. Lysates were transferred to 1.5- or 2-mL tubes and 1 mL of FA buffer (50 mM 4-(2-hydroxyethyl)-1-piperazineethanesulfonic acid-KOH (HEPES-KOH), pH 7.5; 140 mM NaCl; 1 mM EDTA; 1% Triton X-100; 0.1% Na-deoxycholate; protease inhibitor cocktail EDTA-free) was added. The mixture was centrifuged for 1 min at 20,817 x g at 4°, and the pellet was washed once more with FA buffer.

The pellet was resuspended in 450 µL of FA, divided over two 1.5-mL tubes, and sonicated for 6−7 min in a Bioruptor (Diagenode) with 30 sec on-off cycles on high power. Lysates were cleared by centrifugation for 5 min at 4° at 20,817 x g. Supernatant containing chromatin was transferred to 1.5-mL tube and 1 mL of FA was added to samples of the 12-mL tubes. Screw-cap tube chromatin samples were treated with micrococcal nuclease to generate mononucleosomes. For these samples, 800 µL of final buffer (15 mM Tris, pH 7.4; 50 mM NaCl; 1.5 mM CaCl_2_; 5 mM β-mercaptoethanol; 5 mM MgCl_2_) was added. Samples were incubated with 30 units of micrococcal nuclease (Fermentas) at 37° for 20 min. The reaction was stopped by adding ethylene glycol tetraacetic (EGTA) acid and EDTA to a final concentration of 10 mM and placing tubes on ice. The chromatin solution was centrifuged for 15 min at 20,817 x g at 4°; the supernatant was transferred to a new 1.5-mL tube and stored at −20°.

Magnetic dynabeads coupled with Protein G (Dynal) were incubated in PBS containing 5 mg/mL bovine serum albumin (BSA) with antibody for at least 4 hr at 4°. Then, 200 µL of soluble chromatin was added to prepared dynabeads and incubated rotating overnight at 4° and 1 mL of FA buffer was added and samples were incubated rotating for 5 min at room temperature. The samples were washed twice with each of the buffers FA, FA-HS (50 mM HEPES-KOH, pH 7.5; 500 mM NaCl; 1 mM EDTA; 1% Triton X-100; 0.1% Na-deoxycholate), RIPA (10 mM Tris, pH 8; 250 mM LiCl; 0.5% NP-40; 0.5% Na-deoxycholate; 1 mM EDTA). Finally, the samples were washed once with TE (10 mM Tris, pH 8; 1 mM EDTA). Then 100 µL of elution buffer (50 mM Tris, pH 8; 10 mM EDTA; 1% SDS) was added to the samples and incubated for 10 min at 65°. Subsequently the samples were centrifuged 1 min at 20,817 x g and 80 µL of supernatant was collected. Then, 70 µL of TE was added to samples and cross links were reversed in 0.625 mg/mL ProtK and 3 µg/mL RNaseA incubated for 1 hr at 50° and subsequently overnight at 65°. For input samples, 40 µL of chromatin solution was combined with 60 µl elution buffer and 70 µL of TE and treated in the same manner as IP samples to reverse cross links. DNA was purified by using the High Pure PCR Product Purification Kit (Roche). Alternatively, DNA was extracted by using Chelex-100 resin (Bio-Rad) (Walsh *et al.* 1991; Nelson *et al.* 2009).

### Quantitative PCR

Quantitative real time-PCR (qPCR) was performed with SYBRgreen master mix (Applied Biosystems or Roche) according to the manufacturer’s manual. IP samples were diluted 10 times, and input samples were diluted 100 times before analyzing by qPCR on a 7500 Fast Real-Time PCR system (Applied Biosystems) or LightCycler 480 II (Roche). qPCR primers are shown in Table 3.

**Table 3:** Primer sequences used for qPCR

Primer	Sequence
PTC6_Qfor	ATCGGGGCAATTAAGCATC
PTC6_Qrev	CCCGTAACAAGTCCAGCTTC
RSA4_Qfor	TCTCTGGGAAGTTGAGCCTCTT
RSA4_Qrev	CACAATATACAGATGTCCACCCTGAT
SPA2_Qfor	ATCAAGAGAAGAGGGTTCGACAAG
SPA2_Qrev	CATCGGCTGCGGTAATGG

qPCR, quantitative real-time polymerase chain reaction.

### Microscopy

Microscopy samples were fixed with 4% formaldehyde, stained with 1 μg/mL Hoechst 33342 (Invitrogen) and mounted with Vectashield solution (Vector Laboratories) onto concanavalin A−coated cover slides. Samples were analyzed on a Leica SP5 confocal system using a 405-nm laser to excite Hoechst, 488 nm for yEGFP and 561 nm for mRFP.

## Results and Discussion

### RITE cassette and Cre vector construction

RITE cassettes containing different combinations of small epitope tags or fluorescent tags were generated by combining tag modules of previously described RITE cassettes or with new epitope tags as described in the section *Materials and Methods*. The RITE cassettes are shown in Figure 2A. Many of the RITE cassettes contain unique restriction sites between the different elements, which facilitates modification of the constructs for other applications. Some of the RITE cassettes contain an invariant tag for simultaneous detection of old and new protein using commercially available antibodies. The cassettes can be integrated by homologous recombination behind any gene of interest (Figure 2B).

**Figure 3:**
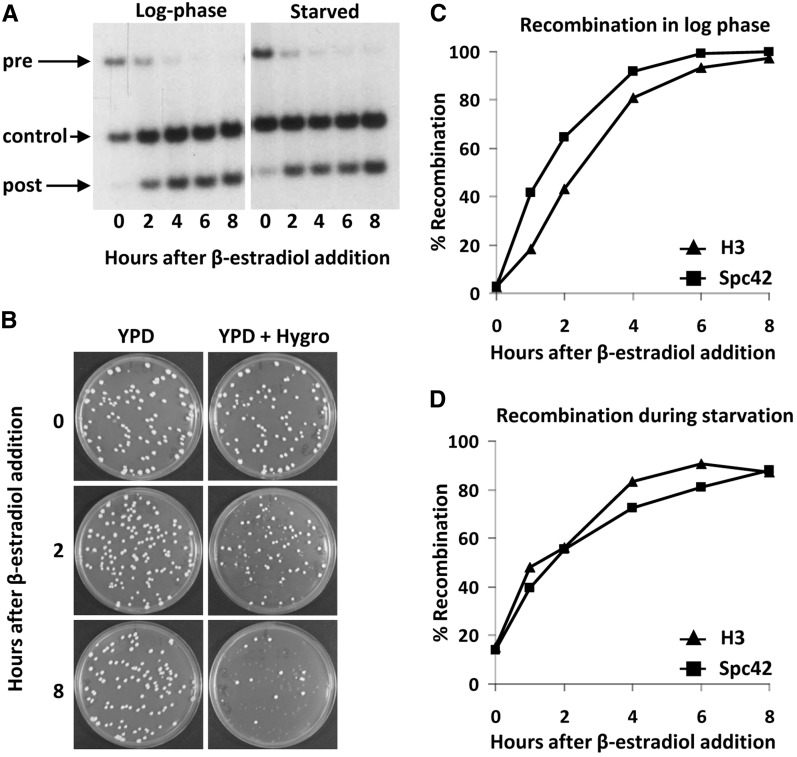
Cre recombination kinetics. (A) Southern blot analysis of H3-HA→T7 (strain NKI2048). Recombination was induced in log-phase or nutrient-starved cells and analyzed using an H3-specific probe on *Hin*dIII-digested DNA. The three bands recognized are specific for preswitch (pre), postswitch (post), or an internal control present in both (control). (B) Plating assay of starved Spc42-3xHA→3xT7-RFP cells (strain SPC42R) after induction of Cre-recombination. Cells were plated on YEPD (YPD) media and then replica plated to YEPD media containing Hygromycin (Hygro). Quantification of recombination levels as measured by plating assay of H3-V5→HA-6xHIS (strain NKI8050) and strain SPC42R in log phase (C) and during starvation (D).

Two vectors are available for integration of the hormone-dependent Cre recombinase (Figure 2C). Unique restriction sites in the Cre vectors allow integration of the constructs by single crossover at the *CYC1* terminator region, or at the *HIS3* or *URA3* regions. These options and the efficiency of integration depend on the auxotrophic alleles present in the target strain. For example, targeting of the *HIS3*-Cre cassette (pTW040) to the commonly used *his3Δ200* allele (see http://wiki.yeastgenome.org/index.php/Strains) is inefficient because of the relatively short region of homology on one end. The advantage of targeting to this locus is that the integrated Cre is less prone to be lost by pop-out recombination. When other strategies are used it is recommended to maintain selection for the integrated Cre vector to select against pop-out events. Cre recombinase is expressed constitutively under control of the *TDH3* promoter and *CYC1* terminator and fused to the human Estrogen Binding Domain (EBD), which makes the nuclear activity of Cre dependent on the hormone β-estradiol (Logie and Stewart 1995). Estradiol releases the EBD from cytosolic heat shock proteins and allows entry of the EBD-Cre chimeric protein into the nucleus for recombination (Logie and Stewart 1995). This allows timed control of the RITE-tag switching. Here we use a derivative of Cre-EBD (Cre-EBD78) that is tightly dependent on the addition of β-estradiol in budding yeast (Lindstrom and Gottschling 2009; Dymond *et al.* 2011). Background recombination before induction of the tag switch and recombination efficiency after induction can be determined by Southern blot analysis or by plating cells on non-selective media and then replica-plating the colonies to media containing Hygromycin (see Figure 3 and Verzijlbergen *et al.* 2010). In a typical experiment, the average background recombination is 10% or less, whereas the Cre-induced recombination efficiency is 95% or more (see Figure 3 and Verzijlbergen *et al.* 2010). The completion of a recombination-induced tag switch in a population of cells generally takes a few hours (Figure 3). This time window should be taken into consideration when RITE is applied to study the dynamics of proteins with a very high turnover rate. Of note, the rate of induced *vs.* background recombination can vary between strains, tagged genes, cell cycle stages, and experimental conditions. We occasionally encountered strains that showed high background recombination or low levels of induced recombination (data not shown), the reasons of which are unknown. We also found low levels of induced recombination in synthetic media containing monosodium glutamate instead of ammonium sulfate as the nitrogen source (data not shown).

### Immunoblot detection of RITE tags

**Figure 4:**
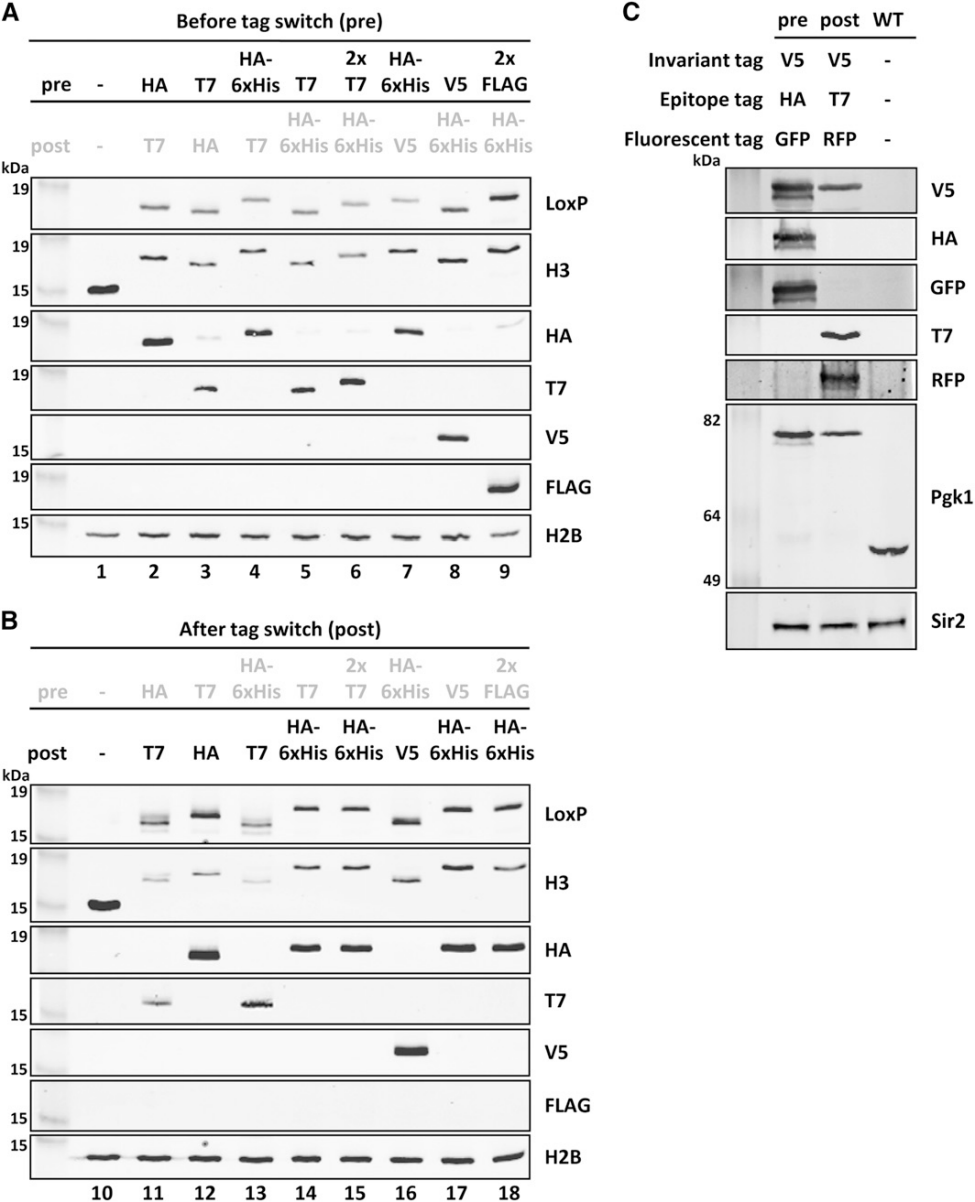
Immunoblot analysis of RITE-tagged histone H3 and Pgk1. (A) Histone H3 (*HHT2*) was tagged with different RITE cassettes. Before tag switch (pre) detects the old Tag 1. A wild-type strain (−, untagged H3) was used as a negative control. H2B was used as a loading control. (B) After tag switch (post) detects the new Tag 2. Postswitch strains are the recombined counterparts of the preswitch strains shown in (A). Strains used: 1. NKI8001; 2. NKI2148; 3. NKI2158; 4. NKI2178; 5. NKI2220; 6. NKI8051; 7. NKI8056; 8. NKI8050; 9. NKI8052; 10. NKI8001; 11. NKI8085; 12. NKI4138; 13. NKI8086; 14. NKI8037; 15. NKI8089; 16. NKI8058; 17. NKI8087; 18. NKI8088. (C) Pgk1 was tagged with an HA-yEGFP→T7-mRFP RITE cassette containing an invariant epitope tag V5 (V5i). Sir2 was used as loading control.

To demonstrate the functionality of the short biochemical epitope tags in the new RITE cassettes, the respective RITE cassettes were PCR amplified and targeted to the histone H3 gene *HHT2* (Figure 2B). To avoid interference of nontagged histone H3, the other gene encoding H3 (*HHT1*) was deleted. We analyzed strains prior to the tag switch as well as strains that had undergone a permanent tag switch. Immunoblot analysis of whole-cell extracts showed that both tags (preswitch shows old Tag 1, postswitch shows new Tag 2) can be detected with the use of tag-specific antibodies (Figure 4, A and B). The old Tag 1 was detected before the switch, whereas the new Tag 2 was detected after the switch. Due to background recombination, low levels of the new Tag 2 were detected before the switch in some of the samples. RITE was also applied to 3-phosphoglycerate kinase (Pgk1), a housekeeping protein. In this case, the RITE cassette contained combinations of short epitope tags and fluorescent tags (pKV015). The old and new tags could be detected on immunoblots before and after the switch, respectively (Figure 4C). In addition, the RITE cassette also harbors an invariant V5 tag, which should be present pre-switch as well as postswitch. Pgk1 containing the invariant V5 tag could be detected in both samples (Figure 4C).

### Detection of RITE tags by ChIP

**Figure 5:**
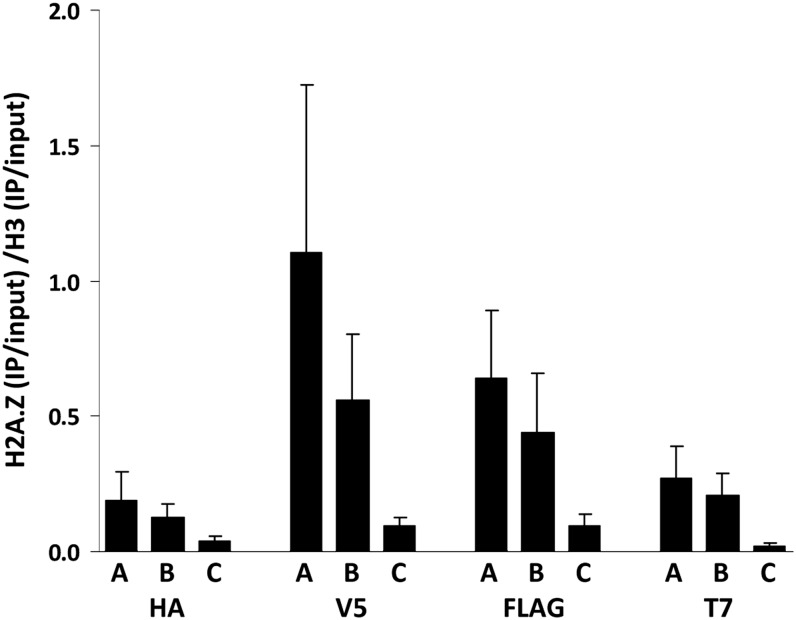
Validation of RITE tags in ChIP assays. RITE-ChIP of H3 and H2A.Z followed by qPCR analysis of three loci: A = *PTC6*, B = *RSA4*, C = *SPA2*. The regions around the transcription start sites of *PTC6* and *RSA4* have high H2A.Z occupancy; the *SPA2* region is located at the 5′ end of the *SPA2* coding sequence and has low H2A.Z occupancy (primer sequences are listed in Table 3). ChIP signals were normalized over the corresponding inputs. The relative ChIP efficiency (H2A.Z/H3 on each of the three loci examined) varied between the four epitope tags. Average of three biological replicates ± SEM. Results were obtained from: HA-ChIP on strains NKI2178 (H3-HA-6xHIS→T7) and NKI8030 (H2A.Z-HA-6xHIS→T7), V5-ChIP on strains NKI8050 (H3-V5→HA-6xHIS) and NKI8053 (H2A.Z-V5→HA-6xHIS), FLAG-ChIP on strains NKI8052 (H3-2xFLAG→HA-6xHIS) and NKI8055 (H2A.Z-2xFLAG→HA-6xHIS), and T7-ChIP on strains NKI8051 (H3-2xT7→HA-6xHIS) and NKI8054 (H2A.Z-2xT7→HA-6xHIS.

One of the advantages of RITE compared with other methods for measuring protein dynamics is the possibility of applying it to affinity purification. We determined how the various tags in the RITE cassettes perform in ChIP experiments. For this purpose, we tagged histone H3 (*HHT2*, in the absence of *HHT1*) and histone variant H2A.Z (*HTZ1*) with different RITE cassettes and performed ChIP-qPCR experiments on preswitch strains (see *Materials and Methods*). Three loci were examined (primer sequences are listed in Table 3), representing high (*PTC6*, *RSA4*) and low (*SPA2*) levels of steady-state H2A.Z and similar levels of H3 (Kobor *et al.* 2004; Albert *et al.* 2007). Indeed, using the RITE tags, we found that H2A.Z levels relative to H3 levels differed according to the previously shown steady-state levels (Figure 5). Note that although the trends were similar, the relative ChIP efficiency (H2A.Z/H3) was not the same for the different RITE tags (Figure 5). Apparently, the short epitope tags do not work equally well for each protein. Fortunately, the RITE assay is very flexible. The expanded RITE cassette series that we describe here affords the selection of epitope tag pairs that work efficiently for the protein of interest. Furthermore, because the RITE cassettes are modular by design, other tag sequences can be readily incorporated.

### Combining RITE with fluorescence microscopy

**Figure 6:**
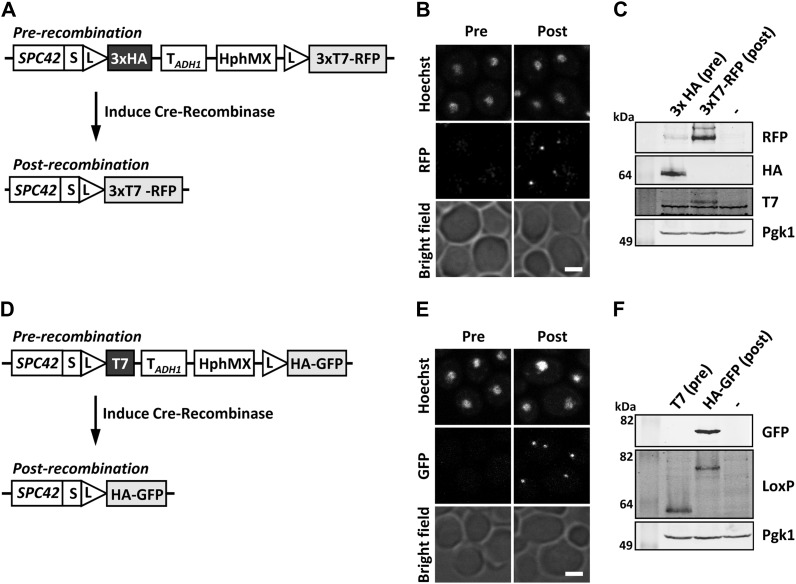
Analysis of fluorescent knock-in RITE cassettes. (A) Spc42, a subunit of the spindle pole body, was tagged with a 3xHA→3xT7-mRFP RITE cassette (strain Spc42R). (B) Fluorescent microscopy of strain Spc42R. In the preswitch sample, no mRFP was detected; in the postswitch sample Spc42-mRFP was clearly visible. (C) Immunoblot analysis of the Spc42R samples described in (B). Pgk1 was used as loading control. (D−F) as in (A−C) but using Spc42 tagged with a 1xT7→1xHA-yEGFP RITE cassette (strain Spc42G). Spc42 containing a single HA or T7 tag could not be detected on blots using the tag-specific antibodies. However, an antibody against spacer-LoxP could visualize old and new Spc42. Scale bars, 2 μm.

The addition of fluorescent markers to the RITE technology adds the possibility of spatiotemporal monitoring of proteins of interest (Verzijlbergen *et al.* 2010; Hotz *et al.* 2012; Menendez-Benito *et al.* 2013). We previously described the HA-yEGFP→T7-mRFP and 3xHA-yEGFP→3xT7-mRFP RITE cassettes (see Figure 2A) (Verzijlbergen *et al.* 2010; Menendez-Benito *et al.* 2013). An mCherry→GFP RITE-like cassette has been described by the Barral lab (Hotz *et al.* 2012). Two additional RITE cassettes can be used to switch from a small epitope tag to a larger fluorescent tag (Figure 2A). They can be used for conditional knock-ins when larger tags cause growth defects. As the protein of interest is only fluorescent after the switch, these RITE cassettes are particularly suited for monitoring synthesis and the spatiotemporal behavior of the new protein by microscopy. Figure 6 shows Spc42, a component of the yeast spindle pole body, tagged with these RITE cassettes (Figure 6, A and D). The spindle-pole body could be observed after the tag switch as a bright dot next to the nucleus (Figure 6, B and E), whereas before the tag switch only background signal was observed. Expression of the proteins was confirmed by immunoblot analysis (Figure 6, C and F). Of note, such color switch tags can be combined with microscopy- or flow cytometry-based genetic screens to identify proteins that control protein or organelle dynamics (Hotz *et al.* 2012).

### Detecting protein dynamics

**Figure 7:**
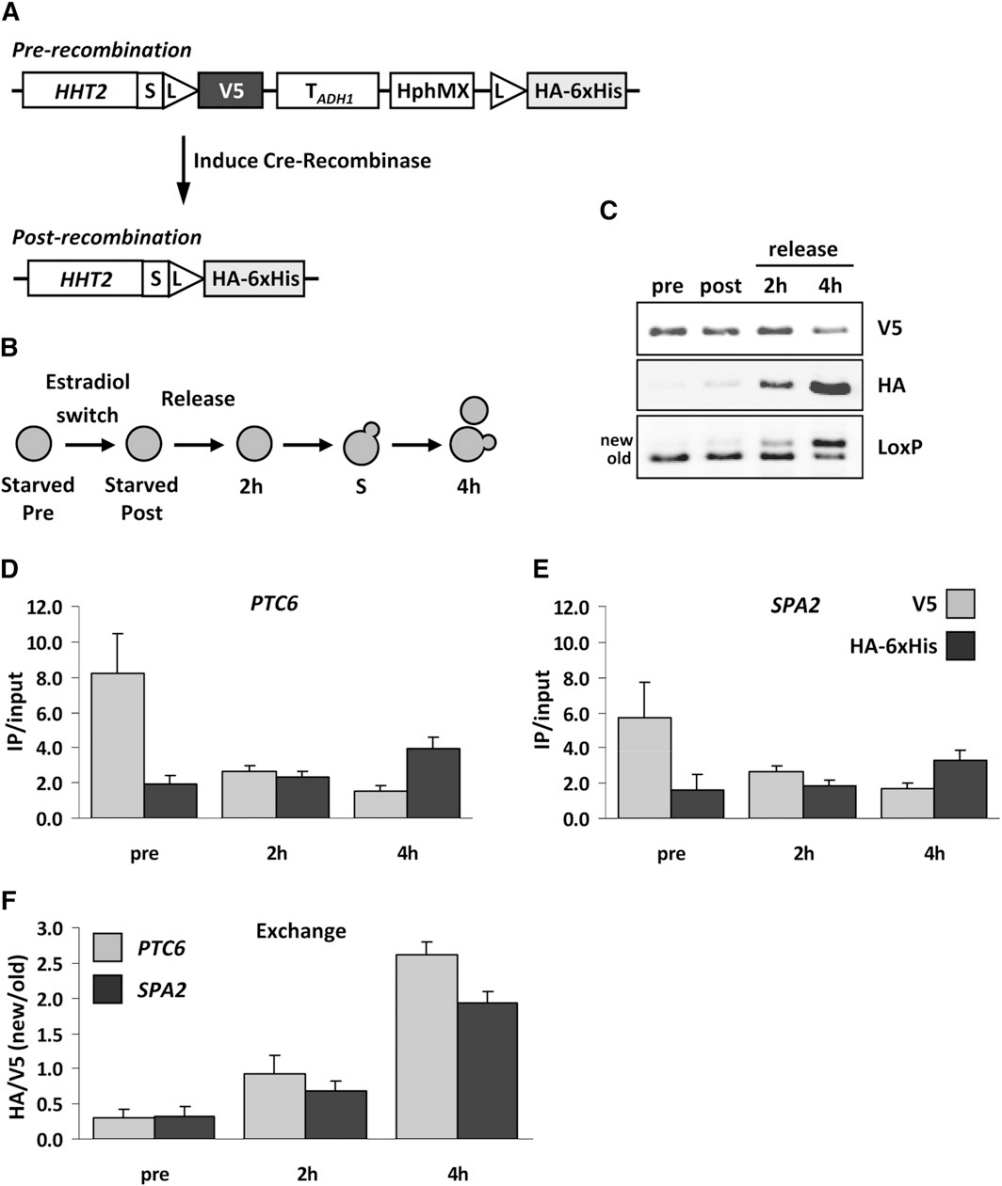
Immunoblot and ChIP analysis of histone H3 dynamics. (A) Histone H3 was tagged with a V5→HA-6xHis RITE cassette in strain NKI8050. (B) Outline of experimental setup. (C) Immunoblot analysis of cells arrested by starvation (pre), switched for 16 hr (post), and subsequently released into fresh media for 2 hr (no cell division) or 4 hr (one cell division). An antibody against spacer-LoxP detects both old and new H3. (D-E) H3-V5 and H3-HA-6xHis ChIP analysis of samples described in (B) to measure occupancy (IP/input) of old and new H3 at *PTC6* (around transcription start site) and *SPA2* (coding sequence) (primer sequences are listed in Table 3). (F) Exchange of histone H3 determined as the ChIP ratio of new H3 over old H3 (HA/V5). Average of three biological replicates ± SEM.

RITE enables the following of protein dynamics under many physiological conditions. Here, we show the application of one of the new RITE cassettes, which switches from V5 to HA-6xHis and was applied to histone H3 (*HHT2*, in the absence of *HHT1*) to measure chromatin protein dynamics (Figure 7A). Cells were arrested by starvation and a preswitch sample was taken before inducing Cre recombinase (Figure 7B). After 16 hr of recombination, a postswitch sample was taken before the cells were released in fresh media. Two additional samples were taken during the release, at 2 hr, before cell division, and at 4 hr, when most of the cells had divided once. During the release, the amount of old Tag 1 (V5) decreased, whereas new Tag 2 (HA-6xHis) increased, as expected (Figure 7C). In the pre- and postswitch samples we detected V5- but hardly any HA-tagged protein. The small amount of HA-tagged protein found in the postswitch sample is consistent with previous results showing that in starved cells there is a low level of histone exchange (Verzijlbergen *et al.* 2010). Using an antibody against the spacer-LoxP peptide sequence, we could detect old Tag 1 and new Tag 2 simultaneously on a single blot due to different mobility on SDS-PAGE gels.

Next, we performed ChIP on these samples and examined occupancy of old and new histone H3 at the *PTC6* gene and the *SPA2* gene (Table 3). For both genes we detected a decrease in V5-tagged protein (old H3) and an increase in HA-tagged protein (new H3) when switched cells were analyzed four hours after release into fresh media (Figure 7, D and E). The level of histone H3 exchange, *i.e.*, the ratio of new H3 over old H3 (new/old), increased over time (Figure 7F). Because the cells are released into log phase and undergo DNA replication, the exchange measured here was mostly caused by replication-coupled histone deposition, and to a lesser extent by replication-independent histone exchange, the latter of which can vary from gene to gene (Dion *et al.* 2007; Jamai *et al.* 2007; Rufiange *et al.* 2007; Deal *et al.* 2010; Verzijlbergen *et al.* 2010).

RITE is a versatile method than can be used to study protein dynamics by different downstream applications on any protein of interest that tolerates C-terminal tags. The GFP tagged yeast library shows the many examples where C-terminal tagging is allowed without significant effects on cell viability or other vital functions (Ghaemmaghami *et al.* 2003; Huh *et al.* 2003). The short epitope tags can be used for immunoblot and affinity purification protocols such as ChIP. Thereby, RITE can be combined with proteomics methods, genomics methods, or DNA-based high-throughput screens (Verzijlbergen *et al.* 2010; De Vos *et al.* 2011; Radman-Livaja *et al.* 2011; Verzijlbergen *et al.* 2011). The fluorescent tags in the RITE cassettes can be applied to measure protein dynamics in single cells by live imaging (Verzijlbergen *et al.* 2010; Hotz *et al.* 2012; Menendez-Benito *et al.* 2013) and further expanded toward high-throughput genetic screening. The suite of RITE cassettes enables a flexible design for many applications. Furthermore, the RITE cassettes are modular by design and can therefore be easily adapted for use in other cell types or organisms. The RITE plasmids and sequence information will be available through EUROSCARF (Frankfurt, http://web.uni-frankfurt.de/fb15/mikro/euroscarf/index.html).

## Supplementary Material

Supporting Information

## References

[bib1] Adams S. R. Tsien R. Y., 2008 Preparation of the membrane-permanent biarsenicals FlAsH-EDT2 and ReAsH-EDT2 for fluorescent labeling of tetracysteine-tagged proteins. Nat. Protoc. 3: 1527–1534 18772880 10.1038/nprot.2008.144 PMC2843588

[bib2] Albert I. Mavrich T. N. Tomsho L. P. Qi J. Zanton S. J., 2007 Translational and rotational settings of H2A.Z nucleosomes across the *Saccharomyces cerevisiae* genome. Nature 446: 572–576 17392789 10.1038/nature05632

[bib3] Brachmann C. B. Davies A. Cost G. J. Caputo E. Li J., 1998 Designer deletion strains derived from *Saccharomyces cerevisiae* S288C: a useful set of strains and plasmids for PCR-mediated gene disruption and other applications. Yeast 14: 115–132 9483801 10.1002/(SICI)1097-0061(19980130)14:2<115::AID-YEA204>3.0.CO;2-2

[bib4] Butko M. T. Yang J. Geng Y. Kim H. J. Jeon N. L., 2012 Fluorescent and photo-oxidizing TimeSTAMP tags track protein fates in light and electron microscopy. Nat. Neurosci. 15: 1742–1751 23103964 10.1038/nn.3246 PMC3509268

[bib5] Deal R. Henikoff S., 2010 a Capturing the dynamic epigenome. Genome Biol. 11: 218 20959022 10.1186/gb-2010-11-10-218 PMC3218653

[bib6] Deal R. B. Henikoff S., 2010 b Catching a glimpse of nucleosome dynamics. Cell Cycle 9: 3389–3390 20703085 10.4161/cc.9.17.13091

[bib7] Deal R. Henikoff J. Henikoff S., 2010 Genome-wide kinetics of nucleosome turnover determined by metabolic labeling of histones. Science 328: 1161–1164 20508129 10.1126/science.1186777 PMC2879085

[bib8] De Vos D. Frederiks F. Terweij M. van Welsem T. Verzijlbergen K. F., 2011 Progressive methylation of ageing histones by Dot1 functions as a timer. EMBO Rep. 12: 956–962 21760613 10.1038/embor.2011.131 PMC3166457

[bib9] Dion M. F. Kaplan T. Kim M. Buratowski S. Friedman N., 2007 Dynamics of replication-independent histone turnover in budding yeast. Science 315: 1405–1408 17347438 10.1126/science.1134053

[bib10] Dymond J. S. Richardson S. M. Coombes C. E. Babatz T. Muller H., 2011 Synthetic chromosome arms function in yeast and generate phenotypic diversity by design. Nature 477: 471–476 21918511 10.1038/nature10403 PMC3774833

[bib11] Ejlassi-Lassallette A. Mocquard E. Arnaud M. C. Thiriet C., 2011 H4 replication-dependent diacetylation and Hat1 promote S-phase chromatin assembly in vivo. Mol. Biol. Cell 22: 245–255 21118997 10.1091/mbc.E10-07-0633 PMC3020919

[bib12] Ghaemmaghami S. Huh W. K. Bower K. Howson R. W. Belle A., 2003 Global analysis of protein expression in yeast. Nature 425: 737–741 14562106 10.1038/nature02046

[bib13] Gietz R. D. Schiestl R. H., 2007 High-efficiency yeast transformation using the LiAc/SS carrier DNA/PEG method. Nat. Protoc. 2: 31–34 17401334 10.1038/nprot.2007.13

[bib14] Hager G. McNally J. Misteli T., 2009 Transcription dynamics. Mol. Cell 35: 741–753 19782025 10.1016/j.molcel.2009.09.005 PMC6326382

[bib15] Hinkson I. Elias J., 2011 The dynamic state of protein turnover: it’s about time. Trends Cell Biol. 21: 292–303 10.1016/j.tcb.2011.02.002 21474317

[bib16] Hotz M. Leisner C. Chen D. Manatschal C. Wegleiter T., 2012 Spindle pole bodies exploit the mitotic exit network in metaphase to drive their age-dependent segregation. Cell 148: 958–972 22385961 10.1016/j.cell.2012.01.041 PMC3779431

[bib17] Huh W. K. Falvo J. V. Gerke L. C. Carroll A. S. Howson R. W., 2003 Global analysis of protein localization in budding yeast. Nature 425: 686–691 14562095 10.1038/nature02026

[bib18] Jamai A. Imoberdorf R. M. Strubin M., 2007 Continuous histone H2B and transcription-dependent histone H3 exchange in yeast cells outside of replication. Mol. Cell 25: 345–355 17289583 10.1016/j.molcel.2007.01.019

[bib19] Jansen L. E. T. Black B. E. Foltz D. R. Cleveland D. W., 2007 Propagation of centromeric chromatin requires exit from mitosis. J. Cell Biol. 176: 795–805 17339380 10.1083/jcb.200701066 PMC2064054

[bib20] Kim H. J. Seol J. H. Han J. W. Youn H. D. Cho E. J., 2007 Histone chaperones regulate histone exchange during transcription. EMBO J. 26: 4467–4474 17914459 10.1038/sj.emboj.7601870 PMC2063486

[bib21] Kobor M. S. Venkatasubrahmanyam S. Meneghini M. D. Gin J. W. Jennings J. L., 2004 A protein complex containing the conserved Swi2/Snf2-related ATPase Swr1p deposits histone variant H2A.Z into euchromatin. PLoS Biol. 2: E131 15045029 10.1371/journal.pbio.0020131 PMC374244

[bib22] Korber P. Luckenbach T. Blaschke D. Horz W., 2004 Evidence for histone eviction in trans upon induction of the yeast PHO5 promoter. Mol. Cell. Biol. 24: 10965–10974 15572697 10.1128/MCB.24.24.10965-10974.2004 PMC533982

[bib23] Lin M. Z. Tsien R. Y., 2010 TimeSTAMP tagging of newly synthesized proteins. Curr. Protoc. Protein Sci. 26. Unit 26: 25 10.1002/0471140864.ps2605s59 PMC2853805 20155731

[bib24] Lindstrom D. L. Gottschling D. E., 2009 The mother enrichment program: a genetic system for facile replicative life span analysis in *Saccharomyces cerevisiae*. Genetics 183: 413–422, 411SI−413SI 19652178 10.1534/genetics.109.106229 PMC2766306

[bib25] Logie C. Stewart A. F., 1995 Ligand-regulated site-specific recombination. Proc. Natl. Acad. Sci. USA 92: 5940–5944 7597057 10.1073/pnas.92.13.5940 PMC41617

[bib26] Menendez-Benito V. van Deventer S. J. Jimenez-Garcia V. Roy-Luzarraga M. van Leeuwen F., 2013 Spatiotemporal analysis of organelle and macromolecular complex inheritance. Proc. Natl. Acad. Sci. USA 110: 175–180 23248297 10.1073/pnas.1207424110 PMC3538235

[bib27] Nelson J. Denisenko O. Bomsztyk K., 2009 The fast chromatin immunoprecipitation method. Methods Mol. Biol. 567: 45–57 19588084 10.1007/978-1-60327-414-2_3

[bib28] Ouellet J. Barral Y., 2012 Organelle segregation during mitosis: lessons from asymmetrically dividing cells. J. Cell Biol. 196: 305–313 22312002 10.1083/jcb.201102078 PMC3275374

[bib29] Radman-Livaja M. Verzijlbergen K. F. Weiner A. van Welsem T. Friedman N., 2011 Patterns and mechanisms of ancestral histone protein inheritance in budding yeast. PLoS Biol. 9: e1001075 21666805 10.1371/journal.pbio.1001075 PMC3110181

[bib30] Ray-Gallet D. Woolfe A. Vassias I. Pellentz C. Lacoste N., 2011 Dynamics of histone H3 deposition in vivo reveal a nucleosome gap-filling mechanism for H3.3 to maintain chromatin integrity. Mol. Cell 44: 928–941 22195966 10.1016/j.molcel.2011.12.006

[bib31] Rocha N. Kuijl C. van der Kant R. Janssen L. Houben D., 2009 Cholesterol sensor ORP1L contacts the ER protein VAP to control Rab7-RILP-p150 Glued and late endosome positioning. J. Cell Biol. 185: 1209–1225 19564404 10.1083/jcb.200811005 PMC2712958

[bib32] Rufiange A. Jacques P. E. Bhat W. Robert F. Nourani A., 2007 Genome-wide replication-independent histone H3 exchange occurs predominantly at promoters and implicates H3 K56 acetylation and Asf1. Mol. Cell 27: 393–405 17679090 10.1016/j.molcel.2007.07.011

[bib33] Russel D. Lasker K. Phillips J. Schneidman-Duhovny D. Velazquez-Muriel J. A., 2009 The structural dynamics of macromolecular processes. Curr. Opin. Cell Biol. 21: 97–108 19223165 10.1016/j.ceb.2009.01.022 PMC2774249

[bib34] Sweet S. M. Li M. Thomas P. M. Durbin K. R. Kelleher N. L., 2010 Kinetics of re-establishing H3K79 methylation marks in global human chromatin. J. Biol. Chem. 285: 32778–32786 20699226 10.1074/jbc.M110.145094 PMC2963384

[bib35] Thiriet C. Hayes J. J., 2005 Replication-independent core histone dynamics at transcriptionally active loci in vivo. Genes Dev. 19: 677–682 15769942 10.1101/gad.1265205 PMC1065721

[bib36] Tong A. H. Boone C., 2006 Synthetic genetic array analysis in *Saccharomyces cerevisiae*. Methods Mol. Biol. 313: 171–192 16118434 10.1385/1-59259-958-3:171

[bib37] van Leeuwen F. Gottschling D. E., 2002 Assays for gene silencing in yeast. Methods Enzymol. 350: 165–186 12073311 10.1016/s0076-6879(02)50962-9

[bib38] van Leeuwen F. Gafken P. R. Gottschling D. E., 2002 Dot1p modulates silencing in yeast by methylation of the nucleosome core. Cell 109: 745–756 12086673 10.1016/s0092-8674(02)00759-6

[bib39] Verzijlbergen K. F. Menendez-Benito V. van Welsem T. van Deventer S. J. Lindstrom D. L., 2010 Recombination-induced tag exchange to track old and new proteins. Proc. Natl. Acad. Sci. USA 107: 64–68 20018668 10.1073/pnas.0911164107 PMC2806724

[bib40] Verzijlbergen K. F. van Welsem T. Sie D. Lenstra T. L. Turner D. J., 2011 A barcode screen for epigenetic regulators reveals a role for the NuB4/HAT-B histone acetyltransferase complex in histone turnover. PLoS Genet. 7: e1002284 21998594 10.1371/journal.pgen.1002284 PMC3188528

[bib41] Walsh P. S. Metzger D. A. Higuchi R., 1991 Chelex 100 as a medium for simple extraction of DNA for PCR-based typing from forensic material. Biotechniques 10: 506–513 1867860

[bib42] Zee B. M. Levin R. S. Xu B. LeRoy G. Wingreen N. S., 2010 In vivo residue-specific histone methylation dynamics. J. Biol. Chem. 285: 3341–3350 19940157 10.1074/jbc.M109.063784 PMC2823435

