# Supplementary material for: Recombination-Induced Tag Exchange (RITE) Cassette Series to Monitor Protein Dynamics in Saccharomyces cerevisiae
Source: G3 (Bethesda). 2013 Aug 1;3(8):1261–72. doi: 10.1534/g3.113.006213 (PMC3737166; doi:10.1534/g3.113.006213)
Supplement: Supporting Information [file supp_3_8_1261__index.html]

Recombination-Induced Tag Exchange (RITE) Cassette Series to Monitor Protein Dynamics in Saccharomyces cerevisiae — Supporting Information 

# Recombination-Induced Tag Exchange (RITE) Cassette Series to Monitor Protein Dynamics in *Saccharomyces cerevisiae*

## Supporting Information for Terweij *et al.*, 2013

**Files in this Data Supplement:**

- File S1 - Sequences of RITE cassettes (PDF, 193 KB)
