# Supplementary material for: Recombination-Induced Tag Exchange (RITE) Cassette Series to Monitor Protein Dynamics in Saccharomyces cerevisiae
Source: G3 (Bethesda). 2013 Aug 1;3(8):1261–72. doi: 10.1534/g3.113.006213 (PMC3737166; doi:10.1534/g3.113.006213)
Supplement: Supporting Information [file supp_g3.113.006213_006213SI.pdf]

G3 · Genes | Genomes | Genetics

Recombination-Induced Tag Exchange (RITE) cassette series to monitor protein dynamics in *Saccharomyces cerevisiae*

Marit Terweij[*], Tibor van Welsem[*], Sjoerd van Deventer[§], Kitty F. Verzijlbergen[*,1], Victoria Menendez-Benito[§], David Ontoso[†], Pedro San-Segundo[†], Jacques Neefjes[§], Fred van Leeuwen[*]

[*]Division of Gene Regulation and [§]Division of Cell Biology, Netherlands Cancer Institute, Netherlands Proteomics Centre, 1066CX Amsterdam, The Netherlands
[†]Instituto de Biología Funcional y Genómica, Consejo Superior de Investigaciones Científcas and University of Salamanca, 37007 Salamanca, Spain
[1]Present address: Wellcome Trust Centre for Cell Biology, University of Edinburgh, Edinburgh EH9 3JR, UK

DOI: 10.1534/g3.113.006213

File S1

Sequences of RITE cassettes

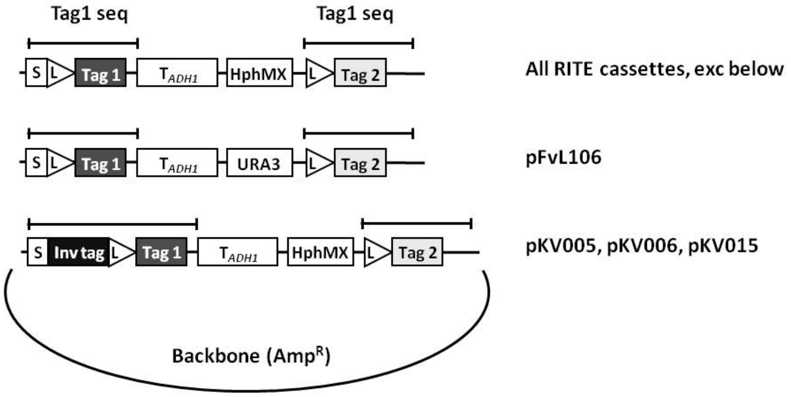

pKV005
>TAG seq1:
GGTGGATCTGGTGGATCTGCTAGCGAACAAAAGTTGATTTCTGAAGAAGATTTGAGCGCTATAACTTCGTATAAT
GTATGCTATACGAAGTTATCAGGTACCTACCCATACGATGTTCCTGACTATGCGACTAGTTAA
>TAG seq2:
TCTAGAATAACTTCGTATAATGTATGCTATACGAAGTTATCAGTCGACATGGCAAGCATGACTGGTGGACAGCAA
ATGGGTTGTACATAAGCGGCCGCGCATGCAAGCTTTTGTTCCCTTTAGTGAGGGTTAATTTCGAGCTTGGCGTAA
TCATGGTCATAGCTGTTTCCTGTGTGAAATTGTTATCCGC

pKV006
>TAG seq1:
GGTGGATCTGGTGGATCTGCTAGCGGTAAGCCTATCCCTAACCCTCTCCTCGGTCTCGATTCTACGAGCGCTATA
ACTTCGTATAATGTATGCTATACGAAGTTATCAGGTACCTACCCATACGATGTTCCTGACTATGCGACTAGTTAA
>TAG seq2:
TCTAGAATAACTTCGTATAATGTATGCTATACGAAGTTATCAGTCGACATGGCAAGCATGACTGGTGGACAGCAA
ATGGGTTGTACATAAGCGGCCGCGCATGCAAGCTTTTGTTCCCTTTAGTGAGGGTTAATTTCGAGCTTGGCGTAA
TCATGGTCATAGCTGTTTCCTGTGTGAAATTGTTATCCGC

pKV014
>TAG seq1:
GGTGGATCTGGTGGATCTATAACTTCGTATAATGTATGCTATACGAAGTTATCAGGTACCTACCCATACGATGTT
CCTGACTATGCGACTAGCTATCCCTATGACGTCCCGGACTATGCAGGATATCCATATGACGTTCCAGATTACGCT
ACTAGTTAA
>TAG seq2:
TCTAGAATAACTTCGTATAATGTATGCTATACGAAGTTATCAGTCGACATGGCAAGCATGACTGGTGGACAGCAA
ATGGGTTGTACTATGGCTTCTATGACAGGAGGTCAACAGATGGGAGGAATGGCCTCAATGACCGGTGGCCAGCAA
ATGGGATGTACAATGGCCTCCTCCGAGGACGTCATCAAGGAGTTCATGCGCTTCAAGGTGCGCATGGAGGGCTCC
GTGAACGGCCACGAGTTCGAGATCGAGGGCGAGGGCGAGGGCCGCCCCTACGAGGGCACCCAGACCGCCAAGCTG
AAGGTGACCAAGGGCGGCCCCCTGCCCTTCGCCTGGGACATCCTGTCCCCTCAGTTCCAGTACGGCTCCAAGGCC
TACGTGAAGCACCCCGCCGACATCCCCGACTACTTGAAGCTGTCCTTCCCCGAGGGCTTCAAGTGGGAGCGCGTG
ATGAACTTCGAGGACGGCGGCGTGGTGACCGTGACCCAGGACTCCTCCCTGCAGGACGGCGAGTTCATCTACAAG
GTGAAGCTGCGCGGCACCAACTTCCCCTCCGACGGCCCCGTAATGCAGAAGAAGACCATGGGCTGGGAGGCCTCC
ACCGAGCGGATGTACCCCGAGGACGGCGCCCTGAAGGGCGAGATCAAGATGAGGCTGAAGCTGAAGGACGGCGGC
CACTACGACGCCGAGGTCAAGACCACCTACATGGCCAAGAAGCCCGTGCAGCTGCCCGGCGCCTACAAGACCGAC
ATCAAGCTGGACATCACCTCCCACAACGAGGACTACACCATCGTGGAACAGTACGAGCGCGCCGAGGGCCGCCAC
TCCACCGGCGCCAAGCTTTTGTTCCCTTTAGTGAGGGTTAATTTCGAGCTTGGCGTAATCATGGTCATAGCTGTT
TCCTGTGTGAAATTGTTATCCGC

pKV015
>TAG seq1:
GGTGGATCTGGTGGATCTGCTAGCGGTAAGCCTATCCCTAACCCTCTCCTCGGTCTCGATTCTACGAGCGCTATA
ACTTCGTATAATGTATGCTATACGAAGTTATCAGGTACCTACCCATACGATGTTCCTGACTATGCGACTAGTATG

TCTAAAGGTGAAGAATTATTCACTGGTGTTGTCCCAATTTTGGTTGAATTAGATGGTGATGTTAATGGTCACAAA
TTTTCTGTCTCCGGTGAAGGTGAAGGTGATGCTACTTACGGTAAATTGACCTTAAAATTTATTTGTACTACTGGT
AAATTGCCAGTTCCATGGCCAACCTTAGTCACTACTTTAACTTATGGTGTTCAATGTTTTTCTAGATACCCAGAT
CATATGAAACAACATGACTTTTTCAAGTCTGCCATGCCAGAAGGTTATGTTCAAGAAAGAACTATTTTTTTCAAA
GATGACGGTAACTACAAGACCAGAGCTGAAGTCAAGTTTGAAGGTGATACCTTAGTTAATAGAATCGAATTAAAA
GGTATTGATTTTAAAGAAGATGGTAACATTTTAGGTCACAAATTGGAATACAACTATAACTCTCACAATGTTTAC
ATCATGGCTGACAAACAAAAGAATGGTATCAAAGTTAACTTCAAAATTAGACACAACATTGAAGATGGTTCTGTT
CAATTAGCTGACCATTATCAACAAAATACTCCAATTGGTGATGGTCCAGTCTTGTTACCAGACAACCATTACTTA
TCCACTCAATCTGCCTTATCCAAAGATCCAAACGAAAAGAGAGACCACATGGTCTTGTTAGAATTTGTTACTGCT
GCTGGTATTACCCATGGTATGGATGAATTGTATAAAACTAGTTAA
>TAG seq2:
TCTAGAATAACTTCGTATAATGTATGCTATACGAAGTTATCAGTCGACATGGCAAGCATGACTGGTGGACAGCAA
ATGGGTTGTACAATGGCCTCCTCCGAGGACGTCATCAAGGAGTTCATGCGCTTCAAGGTGCGCATGGAGGGCTCC
GTGAACGGCCACGAGTTCGAGATCGAGGGCGAGGGCGAGGGCCGCCCCTACGAGGGCACCCAGACCGCCAAGCTG
AAGGTGACCAAGGGCGGCCCCCTGCCCTTCGCCTGGGACATCCTGTCCCCTCAGTTCCAGTACGGCTCCAAGGCC
TACGTGAAGCACCCCGCCGACATCCCCGACTACTTGAAGCTGTCCTTCCCCGAGGGCTTCAAGTGGGAGCGCGTG
ATGAACTTCGAGGACGGCGGCGTGGTGACCGTGACCCAGGACTCCTCCCTGCAGGACGGCGAGTTCATCTACAAG
GTGAAGCTGCGCGGCACCAACTTCCCCTCCGACGGCCCCGTAATGCAGAAGAAGACCATGGGCTGGGAGGCCTCC
ACCGAGCGGATGTACCCCGAGGACGGCGCCCTGAAGGGCGAGATCAAGATGAGGCTGAAGCTGAAGGACGGCGGC
CACTACGACGCCGAGGTCAAGACCACCTACATGGCCAAGAAGCCCGTGCAGCTGCCCGGCGCCTACAAGACCGAC
ATCAAGCTGGACATCACCTCCCACAACGAGGACTACACCATCGTGGAACAGTACGAGCGCGCCGAGGGCCGCCAC
TCCACCGGCGCCAAGCTTTTGTTCCCTTTAGTGAGGGTTAATTTCGAGCTTGGCGTAATCATGGTCATAGCTGTT
TCCTGTGTGAAATTGTTATCCGC

pKV016
>TAG seq1:
GGTGGATCTGGTGGATCTATAACTTCGTATAATGTATGCTATACGAAGTTATCAGGTACCTACCCATACGATGTT
CCTGACTATGCGACTAGCTATCCCTATGACGTCCCGGACTATGCAGGATATCCATATGACGTTCCAGATTACGCT
ACTAGTATGTCTAAAGGTGAAGAATTATTCACTGGTGTTGTCCCAATTTTGGTTGAATTAGATGGTGATGTTAAT
GGTCACAAATTTTCTGTCTCCGGTGAAGGTGAAGGTGATGCTACTTACGGTAAATTGACCTTAAAATTTATTTGT
ACTACTGGTAAATTGCCAGTTCCATGGCCAACCTTAGTCACTACTTTAACTTATGGTGTTCAATGTTTTTCTAGA
TACCCAGATCATATGAAACAACATGACTTTTTCAAGTCTGCCATGCCAGAAGGTTATGTTCAAGAAAGAACTATT
TTTTTCAAAGATGACGGTAACTACAAGACCAGAGCTGAAGTCAAGTTTGAAGGTGATACCTTAGTTAATAGAATC
GAATTAAAAGGTATTGATTTTAAAGAAGATGGTAACATTTTAGGTCACAAATTGGAATACAACTATAACTCTCAC
AATGTTTACATCATGGCTGACAAACAAAAGAATGGTATCAAAGTTAACTTCAAAATTAGACACAACATTGAAGAT
GGTTCTGTTCAATTAGCTGACCATTATCAACAAAATACTCCAATTGGTGATGGTCCAGTCTTGTTACCAGACAAC
CATTACTTATCCACTCAATCTGCCTTATCCAAAGATCCAAACGAAAAGAGAGACCACATGGTCTTGTTAGAATTT
GTTACTGCTGCTGGTATTACCCATGGTATGGATGAATTGTATAAAACTAGTTAA
>TAG seq2:
TCTAGAATAACTTCGTATAATGTATGCTATACGAAGTTATCAGTCGACATGGCAAGCATGACTGGTGGACAGCAA
ATGGGTTGTACTATGGCTTCTATGACAGGAGGTCAACAGATGGGAGGAATGGCCTCAATGACCGGTGGCCAGCAA
ATGGGATGTACAATGGCCTCCTCCGAGGACGTCATCAAGGAGTTCATGCGCTTCAAGGTGCGCATGGAGGGCTCC
GTGAACGGCCACGAGTTCGAGATCGAGGGCGAGGGCGAGGGCCGCCCCTACGAGGGCACCCAGACCGCCAAGCTG
AAGGTGACCAAGGGCGGCCCCCTGCCCTTCGCCTGGGACATCCTGTCCCCTCAGTTCCAGTACGGCTCCAAGGCC
TACGTGAAGCACCCCGCCGACATCCCCGACTACTTGAAGCTGTCCTTCCCCGAGGGCTTCAAGTGGGAGCGCGTG
ATGAACTTCGAGGACGGCGGCGTGGTGACCGTGACCCAGGACTCCTCCCTGCAGGACGGCGAGTTCATCTACAAG
GTGAAGCTGCGCGGCACCAACTTCCCCTCCGACGGCCCCGTAATGCAGAAGAAGACCATGGGCTGGGAGGCCTCC
ACCGAGCGGATGTACCCCGAGGACGGCGCCCTGAAGGGCGAGATCAAGATGAGGCTGAAGCTGAAGGACGGCGGC
CACTACGACGCCGAGGTCAAGACCACCTACATGGCCAAGAAGCCCGTGCAGCTGCCCGGCGCCTACAAGACCGAC
ATCAAGCTGGACATCACCTCCCACAACGAGGACTACACCATCGTGGAACAGTACGAGCGCGCCGAGGGCCGCCAC
TCCACCGGCGCCAAGCTTTTGTTCCCTTTAGTGAGGGTTAATTTCGAGCTTGGCGTAATCATGGTCATAGCTGTT
TCCTGTGTGAAATTGTTATCCGC

pVM013
>TAG seq1:
GGTGGATCTGGTGGATCTATAACTTCGTATAATGTATGCTATACGAAGTTATCAGGTACCATGGCAAGCATGACT
GGTGGACAGCAAATGGGTACTAGTTAA
>TAG seq2:
TCTAGAATAACTTCGTATAATGTATGCTATACGAAGTTATCAGTCGACTACCCATACGATGTTCCTGACTATGCG
TGTACAATGTCTAAAGGTGAAGAATTATTCACTGGTGTTGTCCCAATTTTGGTTGAATTAGATGGTGATGTTAAT
GGTCACAAATTTTCTGTCTCCGGTGAAGGTGAAGGTGATGCTACTTACGGTAAATTGACCTTAAAATTTATTTGT

ACTACTGGTAAATTGCCAGTTCCATGGCCAACCTTAGTCACTACTTTAACTTATGGTGTTCAATGTTTTTCTAGA
TACCCAGATCATATGAAACAACATGACTTTTTCAAGTCTGCCATGCCAGAAGGTTATGTTCAAGAAAGAACTATT
TTTTTCAAAGATGACGGTAACTACAAGACCAGAGCTGAAGTCAAGTTTGAAGGTGATACCTTAGTTAATAGAATC
GAATTAAAAGGTATTGATTTTAAAGAAGATGGTAACATTTTAGGTCACAAATTGGAATACAACTATAACTCTCAC
AATGTTTACATCATGGCTGACAAACAAAAGAATGGTATCAAAGTTAACTTCAAAATTAGACACAACATTGAAGAT
GGTTCTGTTCAATTAGCTGACCATTATCAACAAAATACTCCAATTGGTGATGGTCCAGTCTTGTTACCAGACAAC
CATTACTTATCCACTCAATCTGCCTTATCCAAAGATCCAAACGAAAAGAGAGACCACATGGTCTTGTTAGAATTT
GTTACTGCTGCTGGTATTACCCATGGTATGGATGAATTGTATAAATAAAAAAGCTTTTGTTCCCTTTAGTGAGGG
TTAATTCCGAGCTTGGCGTAATCATGGTCATAGCTGTTTCCTGTGTGAAATTGTTATCCGC

pFVL106
>TAG seq1:
GGTGGATCTGGTGGATCTATAACTTCGTATAATGTATGCTATACGAAGTTATCAGGTACCTACCCATACGATGTT
CCTGACTATGCGACTAGTTAA
>TAG seq2:
TCTAGAATAACTTCGTATAATGTATGCTATACGAAGTTATCAGTCGACATGGCAAGCATGACTGGTGGACAGCAA
ATGGGTTGTACATAAGCGGCCGCGCATGCAAGCTTTTGTTCCCTTTAGTGAGGGTTAATTTCGAGCTTGGCGTAA
TCATGGTCATAGCTGTTTCCTGTGTGAAATTGTTATCCGC

pFVL118
>TAG seq1:
GGTGGATCTGGTGGATCTATAACTTCGTATAATGTATGCTATACGAAGTTATCAGGTACCTACCCATACGATGTT
CCTGACTATGCGACTAGTTAA
>TAG seq2:
TCTAGAATAACTTCGTATAATGTATGCTATACGAAGTTATCAGTCGACATGGCAAGCATGACTGGTGGACAGCAA
ATGGGTTGTACATAAGCGGCCGCGAATTCGAGCTCGGTACCCGGGGATCCTCTAGAGTCGACCTGCAGGCATGCA
AGCTTTTGTTCCCTTTAGTGAGGGTTAATTCCGAGCTTGGCGTAATCATGGTCATAGCTGTTTCCTGTGTGAAAT
TGTTATCCGC

pFVL119
>TAG seq1:
GGTGGATCTGGTGGATCTATAACTTCGTATAATGTATGCTATACGAAGTTATCAGGTACCTACCCATACGATGTT
CCTGACTATGCGACTAGTTAA
>TAG seq2:
TCTAGAATAACTTCGTATAATGTATGCTATACGAAGTTATCAGTCGACATGGCAAGCATGACTGGTGGACAGCAA
ATGGGTTGTACATAAGCGGCCGCGCATGCAAGCTTTTGTTCCCTTTAGTGAGGGTTAATTTCGAGCTTGGCGTAA
TCATGGTCATAGCTGTTTCCTGTGTGAAATTGTTATCCGC

pFVL160
>TAG seq1:
GGTGGATCTGGTGGATCTATAACTTCGTATAATGTATGCTATACGAAGTTATCAGGTACCTACCCATACGATGTT
CCTGACTATGCGACTAGCTATCCCTATGACGTCCCGGACTATGCAGGATATCCATATGACGTTCCAGATTACGCT
ACTAGTTAA
>TAG seq2:
TCTAGAATAACTTCGTATAATGTATGCTATACGAAGTTATCAGTCGACATGGCAAGCATGACTGGTGGACAGCAA
ATGGGTTGTACTATGGCTTCTATGACAGGAGGTCAACAGATGGGAGGAATGGCCTCAATGACCGGTGGCCAGCAA
ATGGGATGTACATAAGCGGCCGCGCATGCAAGCTTTTGTTCCCTTTAGTGAGGGTTAATTTCGAGCTTGGCGTAA
TCATGGTCATAGCTGTTTCCTGTGTGAAATTGTTATCCGC

pTW073
>TAG seq1:
GGTGGATCTGGTGGATCTATAACTTCGTATAATGTATGCTATACGAAGTTATCAGGTACCTACCCATACGATGTT
CCTGACTATGCGACTAGTTAA
>TAG seq2:
TCTAGAATAACTTCGTATAATGTATGCTATACGAAGTTATCAGTCGACGGTAAGCCTATCCCTAACCCTCTCCTC
GGTCTCGATTCTTGTACATAAGCGGCCGCGAATTCAATTCGAGCTCGGTACCCGGGGATCCTCTAGAGTCGACCT
GCAGGCATGCAAGCTTTTGTTCCCTTTAGTGAGGGTTAATTCCGAGCTTGGCGTAATCATGGTCATAGCTGTTTC
CTGTGTGAAATTGTTATCCGC

pTW081

>TAG seq1:
GGTGGATCTGGTGGATCTATAACTTCGTATAATGTATGCTATACGAAGTTATCAGGTACCATGGCAAGCATGACT
GGTGGACAGCAAATGGGTACTAGTTAA
>TAG seq2:
TCTAGAATAACTTCGTATAATGTATGCTATACGAAGTTATCAGTCGACTACCCATACGATGTTCCTGACTATGCG
TGTACATAAGCGGCCGCGAATTCAATTCGAGCTCGGTACCCGGGGATCCTCTAGAGTCGACCTGCAGGCATGCAA
GCTTTTGTTCCCTTTAGTGAGGGTTAATTCCGAGCTTGGCGTAATCATGGTCATAGCTGTTTCCTGTGTGAAATT
GTTATCCGC

pTW087
>TAG seq1:
GGTGGATCTGGTGGATCTATAACTTCGTATAATGTATGCTATACGAAGTTATCAGGTACCTACCCATACGATGTT
CCTGACTATGCGCACCATCATCACCATCACACTAGTTAA
>TAG seq2:
TCTAGAATAACTTCGTATAATGTATGCTATACGAAGTTATCAGTCGACATGGCAAGCATGACTGGTGGACAGCAA
ATGGGTTGTACATAAGCGGCCGCGAATTCAATTCGAGCTCGGTACCCGGGGATCCTCTAGAGTCGACCTGCAGGC
ATGCAAGCTTTTGTTCCCTTTAGTGAGGGTTAATTCCGAGCTTGGCGTAATCATGGTCATAGCTGTTTCCTGTGT
GAAATTGTTATCCGC

pTW094
>TAG seq1:
GGTGGATCTGGTGGATCTATAACTTCGTATAATGTATGCTATACGAAGTTATCAGGTACCATGGCAAGCATGACT
GGTGGACAGCAAATGGGTACTAGTTAA
>TAG seq2:
TCTAGAATAACTTCGTATAATGTATGCTATACGAAGTTATCAGGAACTTACCCATACGATGTTCCTGACTATGCG
CACCATCATCACCATCACACTAGTTAATGTACATAAGCGGCCGCGAATTCGAGCTCGGTACCCGGGGATCCTCTA
GAGTCGACCTGCAGGCATGCAAGCTTTTGTTCCCTTTAGTGAGGGTTAATTCCGAGCTTGGCGTAATCATGGTCA
TAGCTGTTTCCTGTGTGAAATTGTTATCCGC

pMT001
>TAG seq1:
GGTGGATCTGGTGGATCTATAACTTCGTATAATGTATGCTATACGAAGTTATCAGGTACCTACCCATACGATGTT
CCTGACTATGCGCACCATCATCACCATCACACTAGTTAA
>TAG seq2:
TCTAGAATAACTTCGTATAATGTATGCTATACGAAGTTATCAGTCGACGGTAAGCCTATCCCTAACCCTCTCCTC
GGTCTCGATTCTTGTACATAAGCGGCCGCGAATTCAATTCGAGCTCGGTACCCGGGGATCCTCTAGAGTCGACCT
GCAGGCATGCAAGCTTTTGTTCCCTTTAGTGAGGGTTAATTCCGAGCTTGGCGTAATCATGGTCATAGCTGTTTC
CTGTGTGAAATTGTTATCCGC

pMT004
>TAG seq1:
GGTGGATCTGGTGGATCTATAACTTCGTATAATGTATGCTATACGAAGTTATCAGGTACCGGTAAGCCTATCCCT
AACCCTCTCCTCGGTCTCGATTCTACTAGTTAA
>TAG seq2:
TCTAGAATAACTTCGTATAATGTATGCTATACGAAGTTATCAGGAACTTACCCATACGATGTTCCTGACTATGCG
CACCATCATCACCATCACACTAGTTAATGTACATAAGCGGCCGCGAATTCGAGCTCGGTACCCGGGGATCCTCTA
GAGTCGACCTGCAGGCATGCAAGCTTTTGTTCCCTTTAGTGAGGGTTAATTTCGAGCTTGGCGTAATCATGGTCA
TAGCTGTTTCCTGTGTGAAATTGTTATCCGC

pMT005
>TAG seq1:
GGTGGATCTGGTGGATCTATAACTTCGTATAATGTATGCTATACGAAGTTATCAGGTACCATGGCTTCTATGACA
GGAGGTCAACAGATGATGGCAAGCATGACTGGTGGACAGCAAATGACTAGTTAA
>TAG seq2:
TCTAGAATAACTTCGTATAATGTATGCTATACGAAGTTATCAGGAACTTACCCATACGATGTTCCTGACTATGCG
CACCATCATCACCATCACACTAGTTAATGTACATAAGCGGCCGCGAATTCGAGCTCGGTACCCGGGGATCCTCTA
GAGTCGACCTGCAGGCATGCAAGCTTTTGTTCCCTTTAGTGAGGGTTAATTTCGAGCTTGGCGTAATCATGGTCA
TAGCTGTTTCCTGTGTGAAATTGTTATCCGC

pMT006

>TAG seq1:
GGTGGATCTGGTGGATCTATAACTTCGTATAATGTATGCTATACGAAGTTATCAGGTACCGACTACAAGGACGAT
GACGATAAAGATTATAAAGATGACGATGACAAGACTAGTTAA
>TAG seq2:
TCTAGAATAACTTCGTATAATGTATGCTATACGAAGTTATCAGGAACTTACCCATACGATGTTCCTGACTATGCG
CACCATCATCACCATCACACTAGTTAATGTACATAAGCGGCCGCGAATTCGAGCTCGGTACCCGGGGATCCTCTA
GAGTCGACCTGCAGGCATGCAAGCTTTTGTTCCCTTTAGTGAGGGTTAATTTCGAGCTTGGCGTAATCATGGTCA
TAGCTGTTTCCTGTGTGAAATTGTTATCCGC

pMT010
>TAG seq1:
GGTGGATCTGGTGGATCTATAACTTCGTATAATGTATGCTATACGAAGTTATCAGGTACCTACCCATACGATGTT
CCTGACTATGCGACTAGTTAA
>TAG seq2:
TCTAGAATAACTTCGTATAATGTATGCTATACGAAGTTATCAGTCGACGACTACAAGGACGATGACGATAAAGAT
TATAAAGATGACGATGACAAGTGTACATAAGCGGCCGCGCATGCAAGCTTTTGTTCCCTTTAGTGAGGGTTAATT
TCGAGCTTGGCGTAATCATGGTCATAGCTGTTTCCTGTGTGAAATTGTTATCCGC

RITE Plasmid backbone
>pVZ1 (underlined sequences indicate overlap with RITE cassette inserts)
<u>TCCTGTGTGAAATTGTTATCCGC</u>TCACAATTCCACACAACATACGAGCCGGAAGCATAAAGTGTAAAGCCTGGGG
TGCCTAATGAGTGAGCTAACTCACATTAATTGCGTTGCGCTCACTGCCCGCTTTCCAGTCGGGAAACCTGTCGTG
CCAGCTGCATTAATGAATCGGCCAACGCGCGGGGAGAGGCGGTTTGCGTATTGGGCGCTCTTCCGCTTCCTCGCT
CACTGACTCGCTGCGCTCGGTCGTTCGGCTGCGGCGAGCGGTATCAGCTCACTCAAAGGCGGTAATACGGTTATC
CACAGAATCAGGGGATAACGCAGGAAAGAACATGTGAGCAAAAGGCCAGCAAAAGGCCAGGAACCGTAAAAAGGC
CGCGTTGCTGGCGTTTTTCCATAGGCTCCGCCCCCCTGACGAGCATCACAAAAATCGACGCTCAAGTCAGAGGTG
GCGAAACCCGACAGGACTATAAAGATACCAGGCGTTTCCCCCTGGAAGCTCCCTCGTGCGCTCTCCTGTTCCGAC
CCTGCCGCTTACCGGATACCTGTCCGCCTTTCTCCCTTCGGGAAGCGTGGCGCTTTCTCATAGCTCACGCTGTAG
GTATCTCAGTTCGGTGTAGGTCGTTCGCTCCAAGCTGGGCTGTGTGCACGAACCCCCCGTTCAGCCCGACCGCTG
CGCCTTATCCGGTAACTATCGTCTTGAGTCCAACCCGGTAAGACACGACTTATCGCCACTGGCAGCAGCCACTGG
TAACAGGATTAGCAGAGCGAGGTATGTAGGCGGTGCTACAGAGTTCTTGAAGTGGTGGCCTAACTACGGCTACAC
TAGAAGGACAGTATTTGGTATCTGCGCTCTGCTGAAGCCAGTTACCTTCGGAAAAAGAGTTGGTAGCTCTTGATC
CGGCAAACAAACCACCGCTGGTAGCGGTGGTTTTTTTGTTTGCAAGCAGCAGATTACGCGCAGAAAAAAAGGATC
TCAAGAAGATCCTTTGATCTTTTCTACGGGGTCTGACGCTCAGTGGAACGAAAACTCACGTTAAGGGATTTTGGT
CATGAGATTATCAAAAAGGATCTTCACCTAGATCCTTTTAAATTAAAAATGAAGTTTTAAATCAATCTAAAGTAT
ATATGAGTAAACTTGGTCTGACAGTTACCAATGCTTAATCAGTGAGGCACCTATCTCAGCGATCTGTCTATTTCG
TTCATCCATAGTTGCCTGACTCCCCGTCGTGTAGATAACTACGATACGGGAGGGCTTACCATCTGGCCCCAGTGC
TGCAATGATACCGCGAGACCCACGCTCACCGGCTCCAGATTTATCAGCAATAAACCAGCCAGCCGGAAGGGCCGA
GCGCAGAAGTGGTCCTGCAACTTTATCCGCCTCCATCCAGTCTATTAATTGTTGCCGGGAAGCTAGAGTAAGTAG
TTCGCCAGTTAATAGTTTGCGCAACGTTGTTGCCATTGCTACAGGCATCGTGGTGTCACGCTCGTCGTTTGGTAT
GGCTTCATTCAGCTCCGGTTCCCAACGATCAAGGCGAGTTACATGATCCCCCATGTTGTGCAAAAAAGCGGTTAG
CTCCTTCGGTCCTCCGATCGTTGTCAGAAGTAAGTTGGCCGCAGTGTTATCACTCATGGTTATGGCAGCACTGCA
TAATTCTCTTACTGTCATGCCATCCGTAAGATGCTTTTCTGTGACTGGTGAGTACTCAACCAAGTCATTCTGAGA
ATAGTGTATGCGGCGACCGAGTTGCTCTTGCCCGGCGTCAATACGGGATAATACCGCGCCACATAGCAGAACTTT
AAAAGTGCTCATCATTGGAAAACGTTCTTCGGGGCGAAAACTCTCAAGGATCTTACCGCTGTTGAGATCCAGTTC
GATGTAACCCACTCGTGCACCCAACTGATCTTCAGCATCTTTTACTTTCACCAGCGTTTCTGGGTGAGCAAAAAC
AGGAAGGCAAAATGCCGCAAAAAAGGGAATAAGGGCGACACGGAAATGTTGAATACTCATACTCTTCCTTTTTCA
ATATTATTGAAGCATTTATCAGGGTTATTGTCTCATGAGCGGATACATATTTGAATGTATTTAGAAAAATAAACA
AATAGGGGTTCCGCGCACATTTCCCCGAAAAGTGCCACCTGACGTCTAAGAAACCATTATTATCATGACATTAAC
CTATAAAAATAGGCGTATCACGAGGCCCTTTCGTCTCGCGCGTTTCGGTGATGACGGTGAAAACCTCTGACACAT
GCAGCTCCCGGAGACGGTCACAGCTTGTCTGTAAGCGGATGCCGGGAGCAGACAAGCCCGTCAGGGCGCGTCAGC
GGGTGTTGGCGGGTGTCGGGGCTGGCTTAACTATGCGGCATCAGAGCAGATTGTACTGAGAGTGCACCATATGCG
GTGTGAAATACCGCACAGATGCGTAAGGAGAAAATACCGCATCAGGAAATTGTAAACGTTAATATTTTGTTAAAA
TTCGCGTTAAATTTTTGTTAAATCAGCTCATTTTTTAACCAATAGGCCGAAATCGGCAAAATCCCTTATAAATCA
AAAGAATAGACCGAGATAGGGTTGAGTGTTGTTCCAGTTTGGAACAAGAGTCCACTATTAAAGAACGTGGACTCC
AACGTCAAAGGGCGAAAAACCGTCTATCAGGGCGATGGCCCACTACGTGAACCATCACCCTAATCAAGTTTTTTG
GGGTCGAGGTGCCGTAAAGCACTAAATCGGAACCCTAAAGGGAGCCCCCGATTTAGAGCTTGACGGGGAAAGCCG
GCGAACGTGGCGAGAAAGGAAGGGAAGAAAGCGAAAGGAGCGGGCGCTAGGGCGCTGGCAAGTGTAGCGGTCACG
CTGCGCGTAACCACCACACCCGCCGCGCTTAATGCGCCGCTACAGGGCGCGTCGCGCCATTCGCCATTCAGGCTG
CGCAACTGTTGGGAAGGGCGATCGGTGCGGGCCTCTTCGCTATTACGCCAGCTGGCGAAAGGGGGATGTGCTGCA

AGGCGATTAAGTTGGGTAACGCCAGGGTTTTCCCAGTCACGACGTTGTAAAACGACGGCCAGTGAATTGTAATAC
GACTCACTATAGGGCGAATTGGGCCCAATGCATTGGCGCCGCGGCCGC<u>GGTGGATCTGGTGGATCT</u>
